# Molecular insights into the primary nucleation of polymorphic amyloid β dimers in DOPC lipid bilayer membrane

**DOI:** 10.1002/pro.4283

**Published:** 2022-05

**Authors:** Olga Press‐Sandler, Yifat Miller

**Affiliations:** ^1^ Department of Chemistry Ben‐Gurion University of the Negev Be'er Sheva Israel; ^2^ Ilse Katz Institute for Nanoscale Science and Technology, Ben‐Gurion University of the Negev Be'er Sheva Israel

**Keywords:** Alzheimer's disease, amyloids, DOPC membrane, neurodegenerative disease, protein aggregation

## Abstract

Alzheimer's disease (AD) pathology is characterized by loss of memory cognitive and behavioral deterioration. One of the hallmarks of AD is amyloid β (Aβ) plaques in the brain that consists of Aβ oligomers and fibrils. It is accepted that oligomers, particularly dimers, are toxic species that are produced extracellularly and intracellularly in membranes. It is believed that the disruption of membranes by polymorphic Aβ oligomers is the key for the pathology of AD. This is a first study that investigate the effect of polymorphic “α‐helix/random coil” and “fibril‐like” Aβ dimers on 1,2‐dioleoyl‐*sn*‐glycero‐3‐phosphocholine (DOPC) membrane. It has been found that the DOPC membrane promotes Aβ_1–42_ “fibril‐like” dimers and impedes Aβ_1–42_ “α‐helix/random coil” dimers. The N‐termini domains within Aβ_1–42_ dimers play a role in Aβ aggregation in membrane milieus. In addition, the aromatic π–π interactions (involving residues F19 and F20 in Aβ_1–42_) are the driving forces for the hydrophobic interactions that initiate the primary nucleation of polymorphic Aβ_1–42_ dimers within DOPC membrane. Finally, the DOPC bilayer membrane thickness is locally decreased, and it is disrupted by an embedded distinct Aβ_1–42_ dimer, due to relatively large contacts between Aβ_1–42_ monomers and the DOPC membrane. This study reveals insights into the molecular mechanisms by which polymorphic early‐stage Aβ_1–42_ dimers have distinct impacts on DOPC membrane.

**Abbreviations**
AD: Alzheimer's disease
APL: area per lipid
Aβ: amyloid β
CHC: central hydrophobic core
DOPC: 1,2‐dioleoyl‐*sn*‐glycero‐3‐phosphocholine
MD: molecular dynamics
NPT: N‐number of particles, P‐pressure, T‐temperature
NVT: N‐number of particles, V‐volume, T‐temperature
RMSDs: root mean square deviations
SHC: second hydrophobic core
VdW: Van der Waals

## 1 INTRODUCTION

Alzheimer's disease (AD) is a progressive neurodegenerative brain disorder. One of the pathological hallmarks observed in brain of AD patients are senile plaques that are composed of Aβ peptides. It is reported that soluble Aβ oligomers are the primary toxic species in the brain of AD patients.^1^, ^2^ One of the prevailing hypotheses suggests that Aβ peptides can induce toxicity via two disruption mechanisms.^3^ The first mechanism is demonstrated by Aβ oligomers that bind to a membrane surface and form ion channel‐like pores within the membranes. In the second mechanism, the fibrillation of Aβ triggers the membrane fragmentation through a “detergent‐like” mechanism.^3^, ^4^, ^5^ Overall, in these mechanisms the Aβ peptides induce toxicity by altering the biophysical properties of membranes.^6^, ^7^ The toxic Aβ oligomers disrupt the neuronal membranes integrity and increase their permeability.^8^, ^9^, ^10^ The disruptions of the membranes eventually lead to an extensive leakage of ions, particularly Ca^2+^ ions.^10^, ^11^, ^12^ The imbalance of the ions homeostasis ultimately leading to a neuronal dysfunction and cell death.^13^, ^14^ Hence, it is crucial to investigate the interactions between small Aβ oligomers and the membrane. These interactions are the key role to elucidate the toxicity mechanisms that are induced by Aβ oligomers. Therefore, there is a great interest to investigate the molecular basis of the oligomeric assembly pathways,^15^, ^16^ applying high‐resolution NMR.^17^

The interactions between different types of membranes and Aβ trimers,^18^, ^19^, ^20^, ^21^, ^22^, ^23^ or tetramers,^22^, ^24^, ^25^, ^26^, ^27^ or pentamers^28^, ^29^ have been extensively investigated by computational studies. The different sizes of oligomers are expected to present different interactions with membrane. The interactions between the oligomers and the membrane depend on the nature of the residues that are exposed to the membrane surface. Obviously, in the primary nucleation in Aβ aggregation, the smallest oligomers that are produced are the dimers. Moreover, it is suggested that large oligomers are less deleterious than smaller oligomer, and that the dimers are the building blocks for aggregation.^30^, ^31^ Furthermore, the dimers have been isolated from AD brain,^32^ and demonstrated an interruption in the synaptic plasticity and to the impairment of learning and memory.^33^ It has been shown in AD animal models that Aβ dimers reduce the number of synapses.^34^ The fast aggregation of Aβ dimers, and their interactions with monomers, small oligomers, and membranes does not allow for obtaining high‐resolution structures of Aβ dimers.^35^ Moreover, Aβ aggregates are polymorphic both in solution and in membrane milieus, and experimental studies have challenges to solve their structures at the atomic resolution in these milieus. Hence, molecular dynamics (MD) simulations allow to gain insights into the production of polymorphic Aβ dimers and their interactions with membranes at the atomic resolution.

Previously, computational studies examined the binding of Aβ dimers on membrane surfaces,^36^, ^37^, ^38^ and as transmembrane aggregates^28^, ^39^, ^40^, ^41^ on surface of different types of membranes. Some of these studies investigated Aβ dimer fragments, such as Aβ_17–42_ ^28^ and Aβ_29–42_.^39^ A few studies examined the full‐length Aβ_1–40_ dimer,^40^ and Aβ_1–42_ dimer^41^ for only one polymorphic structure. Yet, none of these studies investigated polymorphic states of the full‐length Aβ_1–42_ dimers within a membrane. Moreover, the 1,2‐dioleoyl‐*sn*‐glycero‐3‐phosphocholine (DOPC) bilayer membrane is a common membrane model that is used in experimental studies to mimic neuron's cell.^42^, ^43^ Therefore, the current study provides a first ever study that focuses on polymorphic Aβ_1–42_ dimers within the DOPC membrane.

To date, three important major questions related to Aβ dimerization within DOPC bilayers remained unanswered: (1) What are the formation mechanisms of polymorphic early‐stage Aβ dimers within the membrane environment? (2) What are the interactions between each polymorph of Aβ dimer and the membrane? and (3) What are the effects of the dimers on the properties of the membrane? To address these questions, we examined for the first time four distinct states of Aβ_1–42_ dimers that are inserted into the zwitterionic DOPC bilayer. The first dimer is two “α‐helix/random coil” monomers that are arranged in parallel orientation. The second dimer is two “α‐helix/random coil” monomers that are arranged in antiparallel orientation. These two dimers demonstrate primary nucleation of early‐stage species with non‐β‐strands properties. The third is a parallel “fibril‐like” dimer, and the fourth is an antiparallel “fibril‐like” dimer. These two dimers represent primary nucleation of early‐stage species with β‐strands properties with a structural proximity to fibrils.

Indeed, our simulations show that the DOPC bilayer impedes the aggregation of Aβ_1–42_ “α‐helix/random coil” dimers, but it promotes the initial seeding of Aβ_1–42_ “fibril‐like” dimers. Furthermore, the N‐termini domains within Aβ_1–42_ dimers play role in aggregation. In addition, the aromatic π–π interactions (involving residues F19 and F20) are the driving forces for the hydrophobic interactions that eventually initiate the primary nucleation of polymorphic Aβ_1–42_ dimers within DOPC bilayer. Finally, the DOPC bilayer thickness is locally decreased, and it is disrupted by an embedded distinct Aβ_1–42_ dimer, due to relatively large contacts between Aβ_1–42_ monomers and the DOPC bilayer.

## 2 RESULTS AND DISCUSSION

**FIGURE 1:**
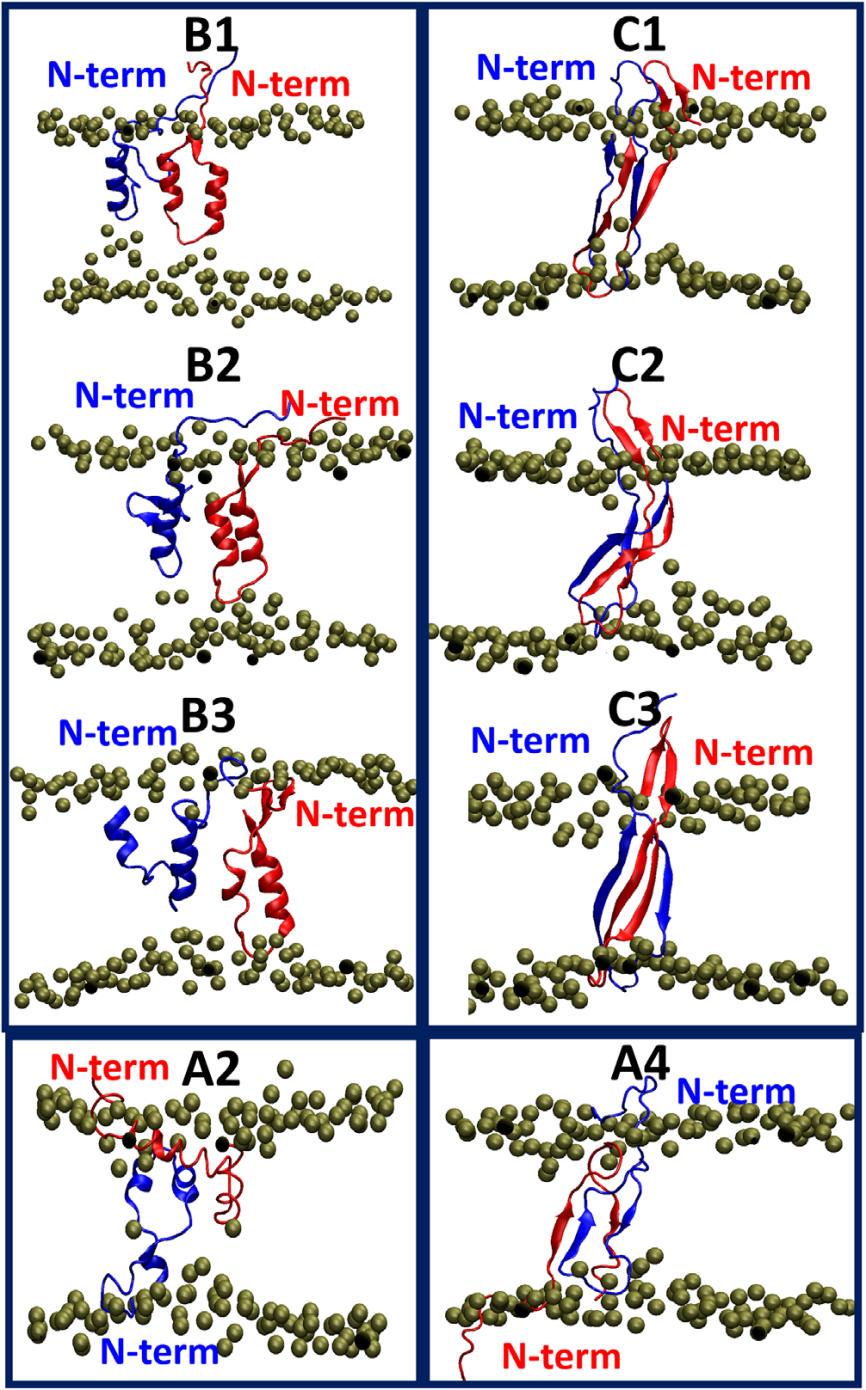
Conformations of Aβ_1–42_ dimers within DOPC bilayer: conformations B1 to B3 were recognized from MD simulations of Model A1, conformation A2 was identified from MD simulations of Model A2, conformations C1 to C3 were established from MD simulations of Model A3, and conformation A4 was obtained from MD simulations of Model A4. Aβ monomers are colored in red and blue. The phosphorus atoms of the DOPC lipid headgroups are shown in Van der Waals spheres. The acyl chains are not shown for clarity

Previously, we examined four polymorphic Aβ_1–42_ dimers in solution milieu^44^: Models A1 and A2—two early‐stage “α‐helix/random coil” dimers—and Models A3 and A4—two “fibril‐like” dimers. Herein, we explored these four polymorphic dimers that are embedded into the zwitterionic DOPC bilayer membrane (Figure S1). The descriptions of the four initial constructed dimer models within the DOPC bilayer are detailed in the Supporting Information. During the MD simulations of these four polymorphic Aβ_1–42_ dimers, a total of eight conformations were recognized (Figure 1): Conformations B1 to B3 were identified along the simulations of Model A1. Simulations of Model A2 revealed conformation A2. Along the simulations of Model A3, three conformations were identified: conformations C1 to C3. Finally, conformation A4 was captured along the simulations of Model A4. The detection of these eight conformations were determined by the root‐mean‐square deviations (RMSDs) and secondary structure analyses for the simulated models (Figures S2–S9) and are detailed in the Supporting Information.

### 2.1 DOPC bilayer impedes the contacts between Aβ monomers within the “α‐helix/random coil” Aβ dimers

**FIGURE 2:**
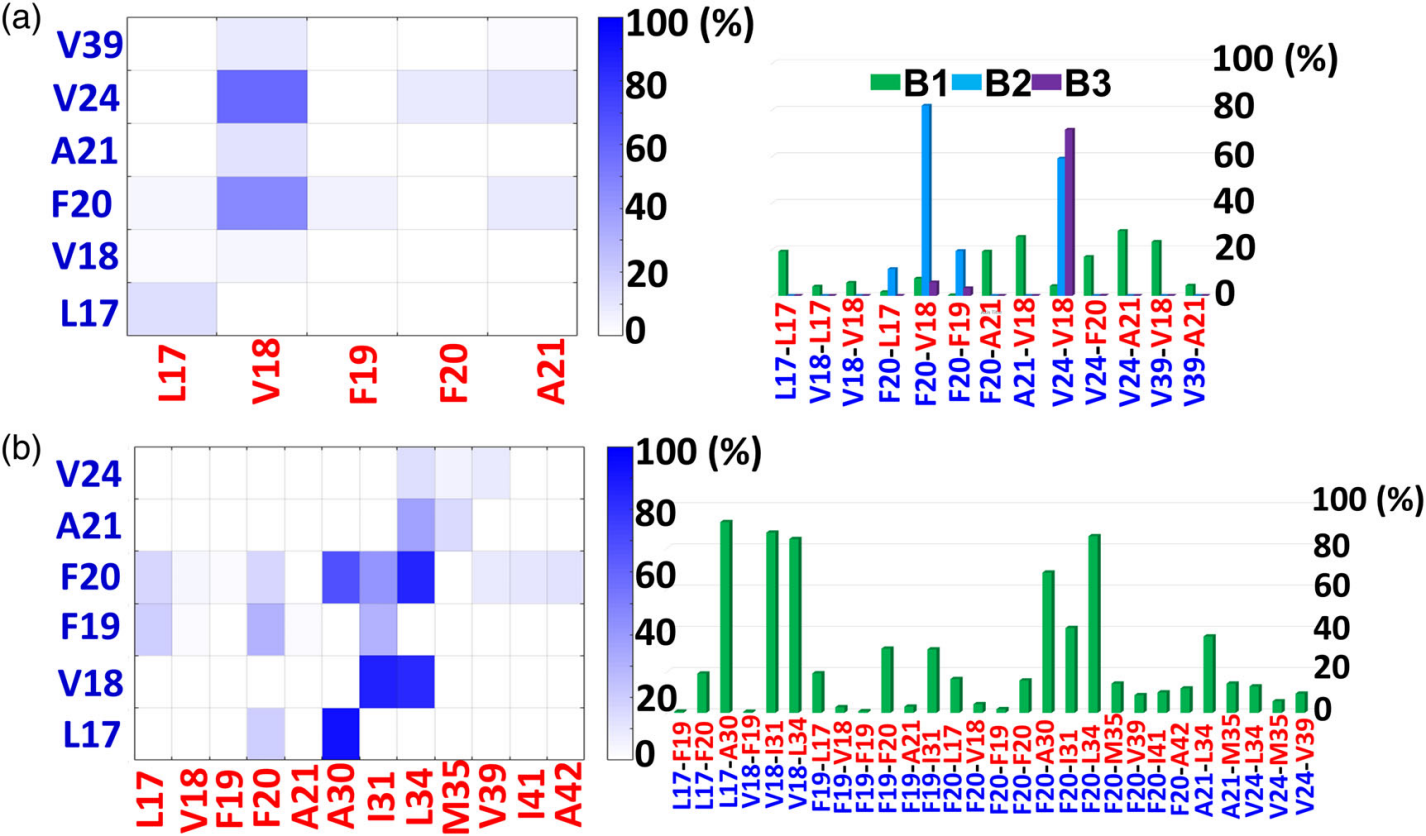
Percentage of hydrophobic interactions between Aβ monomers within the dimer for (a) the total conformations B1 to B3 (left), and for the separated conformation B1 to B3 (right); (b) conformation A2 in two illustrations: a contact map (left) and in a histogram (right). The residues in the contact maps and histograms are colored in red and blue and represent the residues of the two Aβ monomers

It is well‐known that hydrophobic interactions play role in protein–protein interactions in amyloid aggregation.^45^, ^46^ There are three hydrophobic domains within Aβ sequence that are known to contribute for the nucleation of Aβ peptides: the central hydrophobic core (CHC): ^17^LVFF^21^A, the second hydrophobic core (SHC): ^30^AIIGL^35^M, and the C‐terminal domain: ^36^VGGVVI^42^A. Figure 2a represents the hydrophobic interactions between two Aβ monomers within the dimer for the total and the separated conformations B1 to B3. Interestingly, only a few hydrophobic interactions between Aβ monomers play role in the nucleation of Aβ dimerization. In conformation B1, there is a lack of interactions between the monomers within the dimer. In conformations B2 only two contacts (F20‐V18 and V24‐V18) were conserved along the MD simulations, and in conformation B3 only one contact (V24‐V18) was conserved along the MD simulations. Figure 2b demonstrates the hydrophobic interactions between two Aβ monomers within the dimer for conformation A2. In conformation A2, there are more contacts than in conformations B1 to B3. The interactions between the CHC domain of one monomer and the SHC domain of the second monomer were conserved along the MD simulations (Figure 2b).

Previously, we showed that in solution, Aβ monomers within each one of the two “α‐helix/random coil” dimers interact mainly via CHC domains, and in one of these two dimers additional contact domains of the SHC and C termini were involved (Figures S10 and S11).^44^ In the DOPC bilayer, Aβ monomers within the”α‐helix/random coil” dimers (conformations B1–B3 and A2) are less interacted with each other compared within a solution. It is thus suggested that the aggregation pathways of the initial seeding of the “α‐helix/random coil” dimers in solution and in membrane environment are differ. It has been proposed by several experimental studies that the C‐terminal hydrophobic residues play a role in Aβ aggregation in solution milieu, but in membrane environment these residues serve as anchors that bind Aβ peptides to the membrane.^47^ It has been shown by MD simulations of Aβ dimers at different types of membranes (not DOPC bilayer membrane) that the main interactions between the monomers are along the N‐termini domains (D1‐K28).^40^ In the current work, only one electrostatic contact between R5 of one monomer and E11 of the second monomer was identified in the total conformations B1 to B3 in DOPC bilayer (Figure S12). The N‐termini domains contact with the membrane surface, and a few hydrophobic contacts between Aβ monomers at the SHC, C‐termini, and N termini domains were detected. We thus conclude that unlike to the solution milieus, the hydrophobic environment of the acyl chains in the DOPC bilayer impedes the productions of hydrophobic contacts at the CHC, SHC, and C‐termini domains in the “α‐helix/random coil” Aβ dimers. The Aβ peptides are more prone to interact with the membrane core rather than with each other.

### 2.2 DOPC bilayer promotes the primary nucleation of the “fibril‐like” Aβ dimers

**FIGURE 3:**
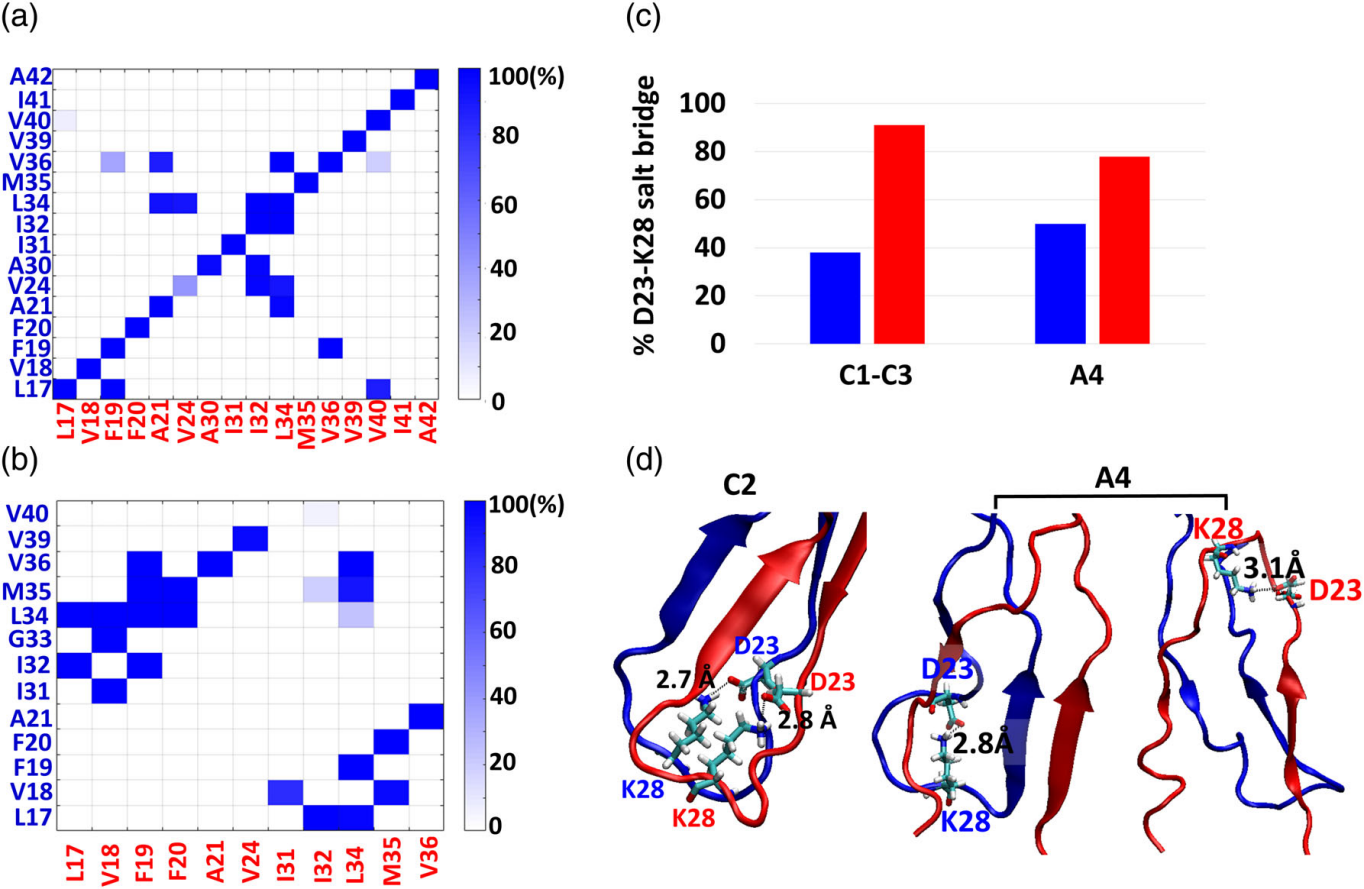
Percentage of hydrophobic interactions between Aβ monomers within the dimer for (a) the total conformations C1 to C3, and (b) conformation A4. The residues in the contact maps are colored in red and blue and represent the residues of the two Aβ monomers. (c) Percentage of intramolecular D23‐K28 salt‐bridge interactions within each Aβ monomer for conformations C1 to C3 and A4. The colored histograms represent the values for each monomer. (d) The measured intramolecular D23‐K28 salt‐bridge interactions in conformations C1 to C3, and A4. The snapshots were taken from the MD simulations

While in the “α‐helix/random coil” dimers (conformations B1–B3 and A2) only a few hydrophobic interactions are presented between the Aβ_1–42_ monomers, in the parallel “fibril‐like” dimers all three nucleation domains (i.e., CHC, SHC, and C‐termini) reveal hydrophobic interactions between the monomers for all total conformations C1 to C3 (Figure 3a). Moreover, for each one of these three conformations C1 to C3, the hydrophobic interactions are displayed at the three nucleation domains (Figure S13). Since these three domains conserved the initial contacts between the monomers within the dimer along the MD simulations, it is proposed that CHC, SHC, and C‐termini domains of Aβ_1–42_ contribute to the initial seeding of the parallel “fibril‐like” dimer within the lipid environment.

In the antiparallel “fibril‐like” conformation A4, the hydrophobic interactions between the CHC domain of one monomer and the SHC domain of the second monomer were conserved along the MD simulations (Figure 3b). These interactions may contribute to the stabilization of the two β‐sheets that “stick together” the two monomers, similarly as we found in the solution.^44^ Hence, we propose that the CHC–SHC contacts between two monomers may be the primary nucleation for the formation of the antiparallel fibrils within the DOPC bilayer.

Previous studies proposed that D23‐K28 salt‐bridge interactions plays important role in the stabilization of polymorphic Aβ fibrils.^48^, ^49^ In one polymorph of Aβ fibril, the D23‐K28 salt‐bridge interactions is recognized as intermolecular interactions between two adjacent peptides,^48^ while in a second polymorph of Aβ fibril, the D23‐K28 salt‐bridge is identified as intramolecular interactions within each peptide.^49^ Herein, the intermolecular D23‐K28 salt‐bridge was not recognized in the parallel and antiparallel Aβ_1–42_ “fibril‐like” dimers. Although the dimer was derived from the fibril that is stabilized by intermolecular D23‐K28 salt‐bridge, intramolecular D23‐K28 salt‐bridge was identified. These interactions were observed more in one monomer than in the second monomer (Figure 3c,d).

The intramolecular salt‐bridge D23‐K28 interactions in the “fibril‐like” dimers correlate with the stabilization of β‐strand properties. In conformations C1 to C3, the monomer that demonstrates above 40% of D23‐K28 salt‐bridge interactions revealed β‐hairpin structure (Figures 3c and S7). In conformation A4, both monomers illustrate above 40% of D23‐K28 salt‐bridge interactions; thus, both monomers reveal similar properties of β‐strands along the sequence (Figures 3c and S9). In summary, these observations emphasize the importance of the D23‐K28 salt‐bridge interactions for the overall stability of the “fibril‐like” dimer within the DOPC bilayer.

Finally, it is essential to investigate the effect of the environment on the stability of Aβ dimers. Previously, we have shown that the β‐strands propensities in both parallel and antiparallel “fibril‐like” Aβ dimers in solution are decreased.^44^ In the current study, the DOPC membrane lipids increase the β‐strands propensities in both “fibril‐like” dimers compared to the solution (Figures S14 and S15). It is thus proposed that the DOPC bilayer serves as a “catalyst” for Aβ dimerization. The DOPC bilayer induces the hydrophobic interactions between the monomers within the “fibril‐like” dimers and stabilizes the dimers by activating the intramolecular D23‐K38 salt‐bridge interactions. The intramolecular hydrophobic interactions were obtained also in simulations of Aβ monomer in membrane.^50^ Therefore, we propose that overall, the DOPC bilayer promotes the primary nucleation of the “fibril‐like” dimers.

### 2.3 Hydrophobic contacts activate aromatic interactions and initiate nucleation and stabilization of Aβ_1–42_ dimers within DOPC bilayer

It was proposed that aromatic π–π interactions are the driving forces for self‐assembly of amyloid fibrils.^51^, ^52^ The diphenylalanine motif, F^19^F^20^, within the CHC domain in Aβ is of a special interest as it was shown to self‐assembled into discrete stiff nanotubes,^53^ while peptides derived from CHC domain completely blocks the formation of fibrils.^54^ The sequence F19‐K28 in Aβ has been shown with high degree of interactions that play a role in elongation of the Aβ fibrils.^55^ The role of this sequence has been also indicated in the lock–dock mechanism of Aβ fibril elongation.^56^ In fact, the removal of the phenylalanine residues prevents amyloid aggregation,^57^ and F19P mutation in Aβ_17–42_ and Aβ_1–42_ channels/barrels prevents their activity.^43^, ^58^ Recently, we have shown that the π–π interactions are essential contacts for the initial seeding of Aβ_1–42_ dimers in the aqueous phase.^44^ Herein, we examined whether these aromatic interactions within Aβ_1–42_ dimers play a role also at lipid membrane environment.

**FIGURE 4:**
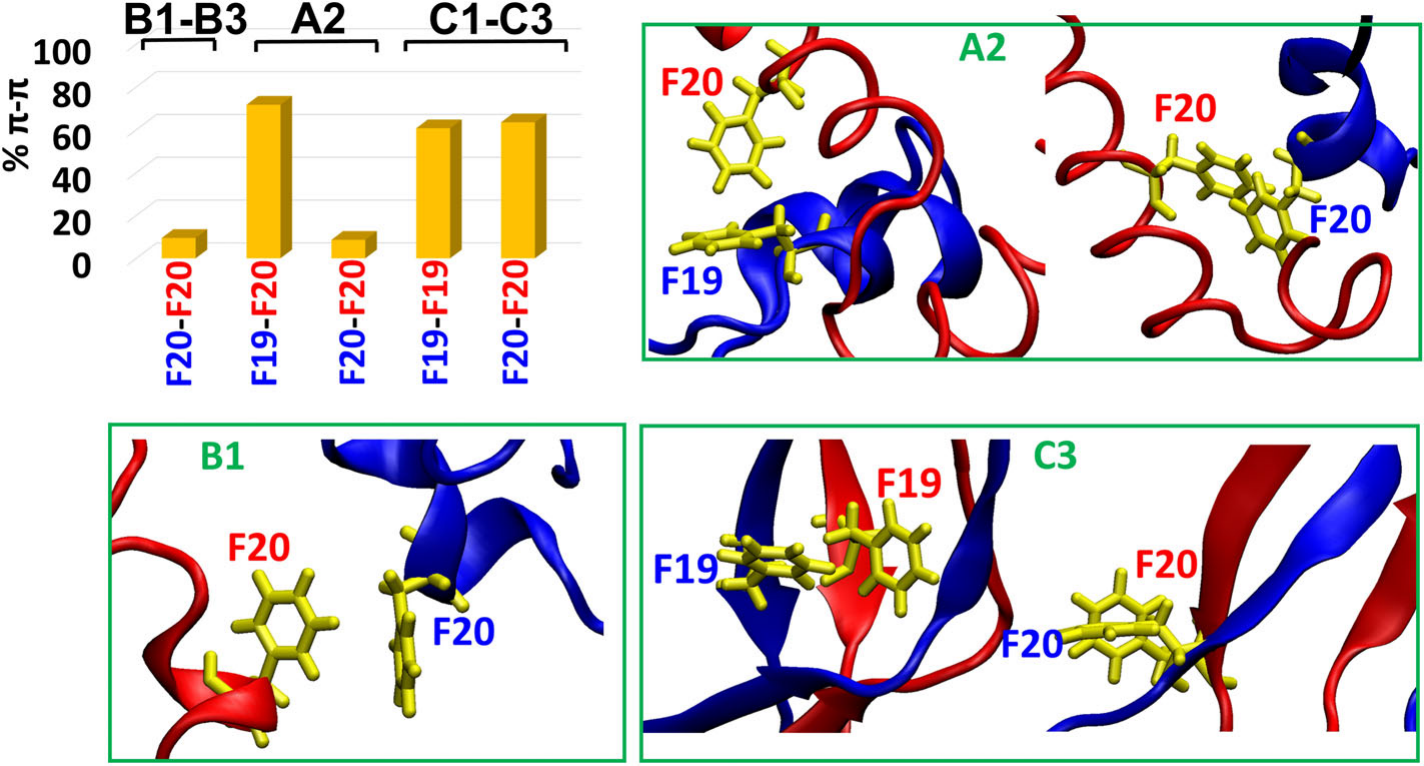
A histogram represents the percentage of aromatic π–π interactions between Aβ monomers for conformations B1 to B3, A2, and C1 to C3. The residues in the histogram are colored in red and blue and represent the residues of the two Aβ monomers. The illustration of the measured aromatic π–π interactions were taken from snapshots of the MD simulations

In the “fibril‐like” parallel dimer (conformations C1–C3), the π–π interactions play a role in stabilization, as seen from the relatively high percentage of the π–π interactions that were identified (Figure 4). Interestingly, the conformational change among the three conformations C1 to C3 does not affect their stabilization by the π–π interactions. This may be attributed to the complete overlap of the hydrophobic interactions within the CHC domains in all these three conformations. Thus, we suggest that hydrophobic contacts activate the aromatic π–π interactions and eventually these interactions stabilize the dimer. In the antiparallel “fibril‐like” dimer (conformation A4), the initial arrangements between the two Aβ monomers do not allow to aromatic interactions, thus these interactions do not play a role in the dimer's stabilization.

In the “α‐helix/random‐coil dimers, the π–π interactions between residues F20 of each monomer were initially integrated in Model A1. In Model A2, the π–π interactions between residues F19 and F20 of each monomer were initially constructed. These π–π interactions were conserved only in conformation A2 (Figure 4) that is derived from Model A2. It is therefore proposed that the conformational change between the three “α‐helix/random coil” conformations B1 to B3 (that are derived from Model A1), and the relatively small number of hydrophobic contacts between the monomers dramatically reduce the aromatic interactions.

### 2.4 DOPC bilayer induces a formation of β‐hairpin along the N‐terminal domain of Aβ_1–42_ dimers

The conformational transition from a α‐helix/random coil structure to β‐sheet rich structures is a fundamental step in amyloid aggregation. Previous experimental studies reported that Aβ adopts β‐structures in the membrane environment.^9^, ^18^, ^59^ An advanced technology using polymeric nanodiscs has shown a trapping of Aβ_1–40_ intermediates with predominantly β‐sheet structures.^60^ Thus, there is an interest to follow the pathways of conformational change from a α‐helix/random coil structure to β‐sheet rich structures of Aβ intermediate aggregates.

In the current study, a transition from a “α‐helix/random coil” to β‐hairpin was presented in one of the monomers along the N‐terminal domain for both “fibril‐like” and “α‐helix/random coil” dimers. In the parallel “fibril‐like” dimer, in all conformations C1 to C3, one monomer conserved the “α‐helix/random coil” along the N‐terminal domain (residues D1‐K16), and the second monomer adopted a stable β‐hairpin structure along the N‐terminal domain (β‐strands along residues A2‐R5 and G9‐V12, and the turn along residues H6‐S8) (Figure S7). In the “α‐helix/random‐coil” dimers, among all three conformations B1 to B3 and conformation A2, only in conformation B3, a short β‐hairpin structure was shown in one of the monomers at the N‐terminal domain (β‐strands along residues F4‐H6 and Y10‐V12, and the turn along residues D7‐G9) (Figure S3). The production of a short β‐hairpin structure at the N‐terminal (residues D1‐Q15) was also presented by MD simulations of Aβ_1–40_ dimer also at other types of membranes.^40^

In conformations B3, and C1 to C3 (that are derived from Models A1 and A3), the β‐hairpin structures were stabilized by the intramolecularly R5‐E11 salt‐bridge interactions (Figure S16). A transition from a “α‐helix/random coil” to β‐hairpin structure at the N‐terminal domain has not been detected in Models A1 and A3 in solution.^44^ Previously, MD simulations of Aβ_1–42_ dimers with initially β‐hairpin structures at the N‐termini of the monomers at zwitterionic dipalmitoylphospatidylcholine (DPPC) membrane showed that the N‐termini of the two monomers conserved the β‐hairpin structures.^41^ Interestingly, it was proposed that the loss of the β‐hairpin structures at the N‐termini decreases Aβ aggregation.^41^ Still, in current study the Aβ_1–42_ dimers were not initially consist of β‐hairpin structure at the N‐termini domains, but the β‐hairpins were detected during the MD simulations. Therefore, we propose that the lipid DOPC environment facilitates the formation of β‐hairpins in the N‐terminal domain of early‐stage dimers at the membrane–solvent interface.

**FIGURE 5:**
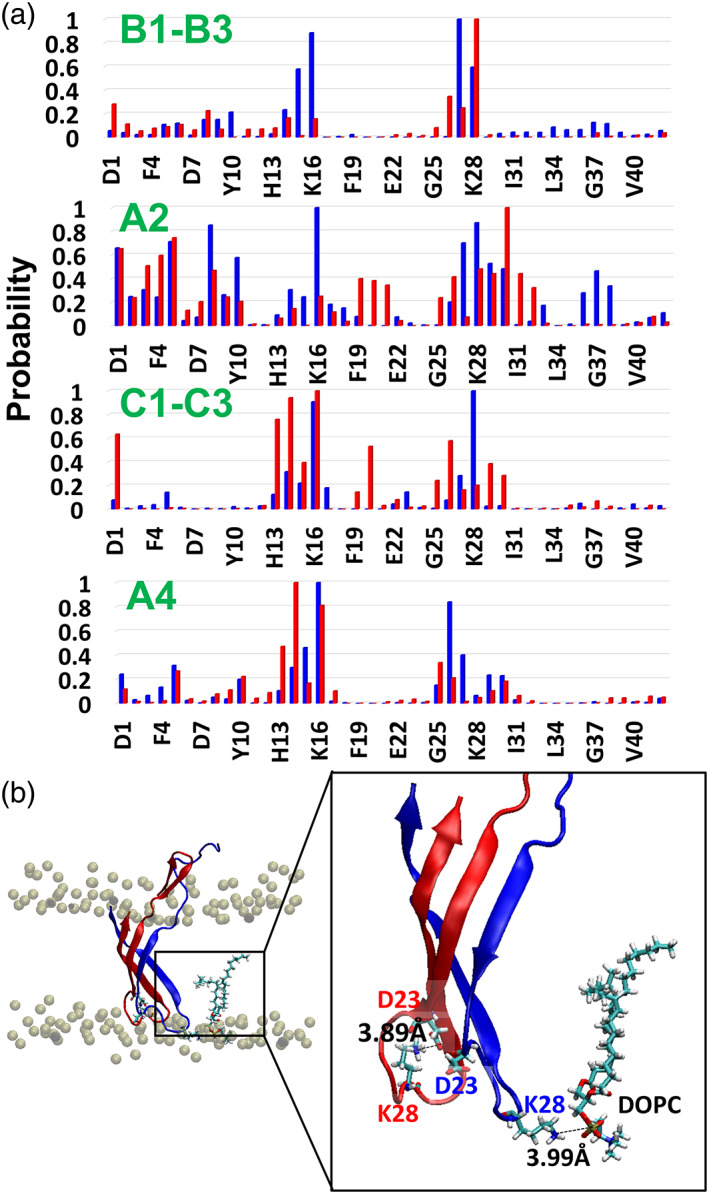
(a) Probability of intermolecular contacts between phosphorus atoms of the DOPC lipids and the nonhydrogen atoms of each Aβ monomer for all eight conformations: B1–B3, A2, C1–C3, and A4. The colored histograms represent the values for each monomer within the dimer; (b) Illustration of salt‐bridge interactions between K28 in one monomer and the DOPC lipid, and intramolecular salt‐bridge interactions between K28 and D23 in the second monomer. The snapshot was taken from MD simulations for conformation C3

The absence of β‐hairpin structures in the other conformations of Aβ_1–42_ dimers (A2 and A4) may be attributed to the relatively large number of contacts between the N‐termini domains with the lipids headgroups that eventually stabilize the dimers at the bilayer surface and prevents the conformational change (Figure 5a). In summary, our results provide for the first time insights into the molecular mechanisms of the transition of the “α‐helix/random coil” to β‐hairpin structure at the N‐terminal domain in two distinct Aβ_1–42_ dimer states within DOPC membrane environment. Moreover, this is a first work that implies the role of N‐terminal domain in the dimerization process of Aβ_1–42_ within DOPC bilayer.

### 2.5 Strong DOPC‐N‐terminal domain interactions prevent a conformational change in antiparallel Aβ dimers

It has been shown by experimental study that early‐stage Aβ oligomers interact with neuronal lipid bilayers and that these interactions induce the toxicity.^3^, ^61^ Therefore, it is important to investigate these specific interactions at the molecular level. Herein, the interactions between residues within each polymorphic Aβ_1–42_ dimers and the DOPC bilayer were investigated at the molecular level. The probability of the interactions between each residue and the headgroup of the DOPC bilayer are presented in Figure 5a. The N‐termini domains (residues D1‐K16) in the antiparallel Aβ_1–42_ dimers (conformations A2 and A4) have relatively more contacts with the lipid headgroups than in the parallel Aβ_1–42_ dimers (conformations B1–B3 and C1–C3). The N‐termini in the antiparallel “α‐helix/random coil” conformation A2 present significantly more contacts with the lipid headgroups than as identified for the parallel “α‐helix/random coil” conformations B1 to B3. The N‐termini in the antiparallel “fibril‐like” conformation A4 illustrate slightly more contacts with the lipid headgroups than have been determined for the parallel “fibril like” conformations C1 to C3. The interactions of the N‐termini domains with the lipid headgroups stabilize Aβ_1–42_ dimer within the DOPC bilayer. The stabilization of the dimer within the DOPC bilayer membrane may avoid a conformational change of the dimer.

Previously, it has been shown that electrostatic interactions between the highly charged N‐terminal domain and residues F20‐A30 with mixed membranes are the driving force for the association of the dimer to the surface of the lipid bilayers.^37^ Herein, residues G25‐A30 in conformations A2, A4, and C1 to C3 interact with the DOPC bilayer. The residues S26‐K28 in conformations B1 to B3 interact with the DOPC bilayer. Our simulations demonstrate that these interactions are revealed for polymorphic Aβ_1–42_ dimer, and not for only one type of Aβ_1–42_ dimer.

In summary, we illustrate a first study that the charged N‐terminal domain and the residues G25‐A30 play a role in the stability of the dimers within the DOPC transmembrane environment. The interactions with the lipids headgroups prevent the conformational change in the antiparallel dimers of the “α‐helix/random coil” conformation A2 and the “fibril‐like” conformation A4. Moreover, the interactions between residues G25‐A30 and the lipids may contribute to the stability and the primary nucleation of the dimer within the DOPC bilayer.

### 2.6 Salt‐bridge interactions between Aβ dimers and DOPC bilayer stabilize Aβ dimers

The residues K16 and K28 interact with the DOPC bilayer lipid headgroups and play an important role in the stabilization of Aβ dimers. The residue K28 forms intramolecularly salt‐bridge interactions with residue D23 and stabilize the fibril‐like dimer. In the parallel dimer conformations C1 to C3, residue K28 in one monomer interacts with the lipid headgroups with relatively high probability, and residues K28 in the second monomer interacts with the lipids headgroups with relatively low probability (Figure 5a). The residue K28 in this second monomer replaces the interaction with the lipid headgroups by the intramolecularly D23‐K28 salt‐bridge interactions in Aβ dimer (Figures 3c,d and 5b).

In the antiparallel conformation A4, the K28 contributes to the intramolecular salt‐bridge D23‐K28 interactions within the dimer (Figure 3c). The residues K28 in each monomer in the antiparallel conformation A4 have a relatively low probability to interact with the lipid headgroups (Figure 5a). This phenomenon implies the importance of the residue K28 to the stability of Aβ_1–42_ dimers within the membrane. Our results are compatible with previous studies that demonstrated the interactions between various types of membranes and K16 or K28 in Aβ_1–40_ monomer,^40^, ^62^ or in Aβ_11–40_ trimer.^20^

### 2.7 Local bilayer thickness of the DOPC is decreased by embedded Aβ_1–42_ dimers

It was shown that Aβ aggregates interact with neuronal membranes and yield to the destabilization of neuronal membranes and to their death.^63^ Several mechanisms have been proposed to illustrate the disruption of membranes by Aβ aggregates. Some studies suggested that the thickness of a membrane is affected by Aβ oligomers that yield to nonselective ion leakage through the low dielectric barrier.^8^, ^64^ Other studies proposed that Aβ pores formation within the membrane disrupts the Ca^2+^ homeostasis.^22^, ^42^, ^63^, ^65^, ^66^, ^67^, ^68^ Thus, to date there is a consensus that the presence of Aβ oligomers within membranes may yield to the disruption of the lipid properties.

Herein, we investigate polymorphic Aβ dimers that are embedded into DOPC membrane bilayer. To examine the effect of each polymorphic Aβ dimer on the DOPC bilayer, the area per lipid (APL) values and the bilayer thicknesses were computed for pure DOPC bilayer and for the DOPC‐embedded with polymorphic Aβ_1–42_ dimers (Table 1).

**TABLE 1:** The averaged values (and the standard deviations) of APL and DOPC bilayer thickness for the pure DOPC and for all conformations that are embedded within the DOPC

	APL (Å^2^)^a^ (*T* = 310 K)		Thickness (Å)^b^ (*T* = 310 K)
	Top	Bottom	
Pure DOPC	68.73 ± 1.07	68.73 ± 1.07	38.51 ± 0.5
B1–B3 in DOPC	65.59 ± 2.1	63.38 ± 1.6	37.09 ± 3.4
A2 in DOPC	60.35 ± 2.3	64.62 ± 2.0	37.65 ± 4.1
C1–C3 in DOPC	64.7 ± 1.8	64.7 ± 1.6	37.9 ± 3.1
A4 in DOPC	65.2 ± 1.7	63.4 ± 2.1	37.8 ± 2.5

^a^ An experimental value^69^ for pure DOPC at 303 K: 67.4 Å^2^. A computational value^70^ for pure DOPC at 310 K: 68.8 ± 1.4 Å^2^.

^b^ Experimental value^69^ for thickness of pure DOPC bilayer at 303 K: 36.7 Å^2^. A computational value^70^ for thickness at 310 K: 38.5 Å^2^.

**FIGURE 6:**
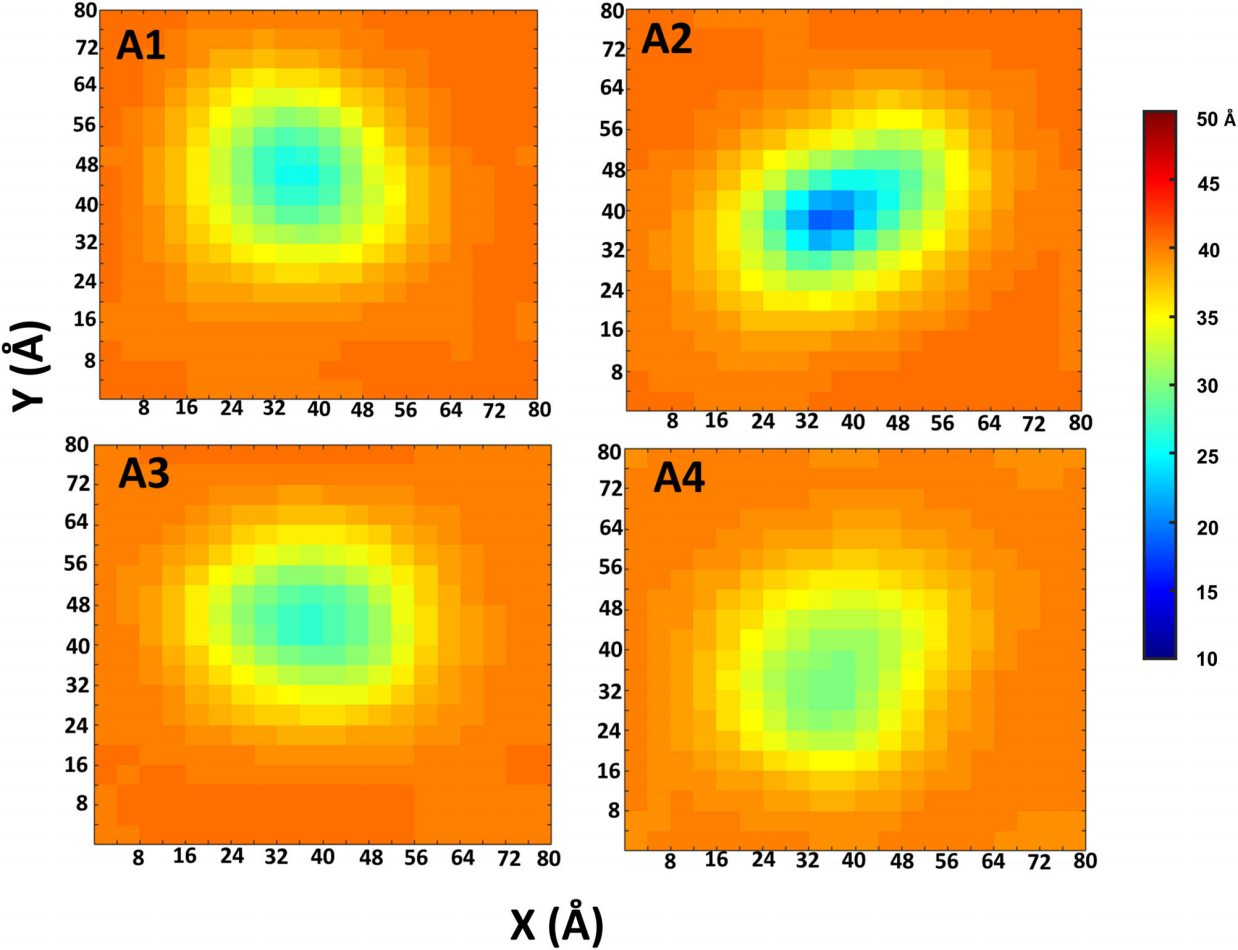
DOPC bilayer thickness distribution maps over the *x*–*y* plane for all Aβ dimer conformations

The APL values for DOPC‐embedded with each one of the dimers are smaller compared to the pure DOPC bilayer. The decrease in the APL values was detected both in the upper and in the lower layers. Although the averaged thickness of the DOPC bilayer is slightly affected by insertion of Aβ_1–42_ dimers compared to the pure DOPC bilayer, the bilayer thickness is locally decreased in the vicinity of Aβ_1–42_ dimers (Figure 6). The most prominent decrease was observed in the “α‐helix/random coil” conformation A2, which demonstrates a local shrinking effect on the membrane. Such a local shrinking effect was previously reported in the MD simulations of Aβ_1–40_ dimer,^40^ Aβ_1–42_ monomers,^26^, ^71^ Aβ_1–42_ tetramer,^26^ and Aβ_1–40_ 24‐mer using coarse‐grained MD simulations.^72^

Herein, we propose that the transmembrane early‐stage Aβ_1–42_ “α‐helix/random coil” dimer conformation A2 adapts a local shrinking of the lower and the upper membrane that eventually yield to negative hydrophobic mismatch. Hydrophobic mismatch is the difference between the hydrophobic thicknesses of the lipid membrane and the transmembrane peptide or protein. Hydrophobic mismatch play a role in stability and function of the transmembrane peptide or protein. Negative hydrophobic mismatch represents thicker lipid membrane and positive hydrophobic mismatch depicts thinner lipid membrane. In the negative hydrophobic mismatch, the hydrophobic part of the transmembrane peptides is too small to match the hydrophobic bilayer thickness. This phenomenon occurs when peptides and proteins aggregates are present in membranes. These peptides or proteins aggregates bind to the membranes via various interactions and re‐organize the lipid bilayer membrane. In the current work, this phenomenon can be attributed to the relatively large number of contacts between Aβ monomers within the dimer conformation A2 and the surrounding phosphate atoms of the lipid headgroups. This may be explained due to electrostatic contacts (Figure 5a) and hydrogen bonds (Figure S17) between the Aβ_1–42_ dimers and the lipid headgroups. In addition, the electrostatic and the Van der Waals (VdW) energies between the DOPC lipids and each monomer within the studied Aβ dimers were computed (Figure S18). The values of the electrostatic and the VdW energies for conformation A2 demonstrated lower energies relatively to the other conformations.

It was reported that the composition of amino acids along the sequence of embedded peptides and proteins in membranes play a crucial role in determining the consequences of hydrophobic mismatch.^73^, ^74^ The amino acids K16 and K28 in the two peptides of conformation A2 were initially embedded into the membrane, and were not tilted on the upper and the lower surfaces of the membranes. During the MD simulations, these amino acids dragged the charged lipid headgroups into the hydrophobic core of the membrane. These actions yield to the relatively strong interactions between the peptides and the headgroups that eventually accelerate to the negative hydrophobic mismatch of the membrane.

## 3 CONCLUSIONS

The interactions between Aβ peptides and lipid membrane depend on the aggregation state and the lipid compositions and may occur by insertion of Aβ peptides.^75^ It is well‐known that Aβ oligomers are the toxic species that lead to the death of neurons. The smallest Aβ oligomers in the primary nucleation are dimers. We previously demonstrated polymorphic Aβ dimers in aqueous solution.^44^ The polymorphic Aβ dimers operate differently in membrane milieu. In the current study, we investigate the molecular mechanisms in which dimers are embedded in DOPC bilayer membrane.

Our study leads to four major conclusions. First, on one hand the DOPC bilayer impedes the primary nucleation of “α‐helix/random coil” Aβ dimers by preventing hydrophobic interactions between Aβ monomers. On the other hand, DOPC bilayer promotes the primary nucleation of Aβ “fibril‐like” dimers by initiating hydrophobic interactions, D23‐K28 salt‐bridge intramolecular interactions within Aβ monomers, and by intermolecular interactions between K28 in Aβ monomer and DOPC membrane. The stabilization of Aβ “fibril‐like” dimers by the DOPC bilayer also occurs due to the formation of β‐strand properties (e.g., a production of β‐hairpins in the hydrophobic core of the DOPC). Second, the hydrophobic interactions between Aβ monomers along the CHC domain of the “fibril‐like” dimer induce the aromatic π–π interactions that are the driving forces in the primary nucleation. The aromatic interactions in the “α‐helix/random coil” Aβ dimers contribute to stable conformations that impede the conformational change. Third, Aβ dimers that have relatively less contacts with the lipid headgroups of the membrane produce β‐hairpin structures along the N‐terminal domain. The lack of production of β‐hairpins along the N‐terminal domain occurs in cases that Aβ have relatively strong and large number of contacts with the lipid headgroups. In these cases, the conformational change is blocked. Fourth, the DOPC bilayer thickness locally decreases by an embedded Aβ dimer, due to relatively large contacts between Aβ monomers and the DOPC bilayer.

Obviously, the lipid composition may affect the nature of the interactions of Aβ dimer with the membrane. Using atomic force microscopy, it was observed that Aβ aggregates interact on the surface in DPPC, and dioleoyl phosphatidyl glycerol membranes, while in DOPC membrane Aβ aggregates are fusing into the lipid membrane.^76^ This experimental study strength our reported findings. This is a first study that illustrates distinct actions of polymorphic Aβ dimers within DOPC membrane at the molecular level. It provides insights into the molecular mechanisms in which polymorphic early‐stage oligomers embedded into the membrane. It is crucial to investigate polymorphic oligomers within membranes because each oligomer operates on the membrane differently than others. Thus, this work may pave the way for future studies to investigate other polymorphic amyloids within different types of membranes. Specifically, future experimental studies using combination of structural biology tools and biophysical techniques are necessary to approve our reported findings. The insights into the molecular mechanisms of the primary nucleation will assist in developing new therapeutic strategies for various amyloid diseases.

## 4 MATERIALS AND METHODS

### 4.1 MD simulations protocol

The all‐atom MD simulations were preformed using the GROMACS‐5.1.4 package.^77^ The all‐atom CHARMM36‐2015 force‐field for GROMACS^78^ was applied to describe the Aβ peptides, lipids, ions, and TIP3P water model.^79^ Each system was first energy minimized by using steepest decent algorithm and then was equilibrated in two phases. Throughout the equilibration, position restrains were applied to all peptides' heavy atoms (i.e., nonhydrogen) with force constant of 1,000 kJ mol^−1^ nm^−2^ in order to adjust the peptides' atoms to lipids and water molecules. In the first phase, the equilibration lasted for 10 ns under isothermal‐isochoric (NVT) conditions by velocity‐rescale thermostat^80^ with coupling constant of 0.1 ps to regulate separately the temperature of 310 K for peptides, lipids, and solvent (including ions). In the second phase, the systems were equilibrated over 50 ns under isothermal‐isobaric (NPT) conditions by using the Nose–Hoover thermostat^81^, ^82^ to regulate the temperature, along with semi‐isotropic Parrinello–Rahman pressure coupling scheme,^83^, ^84^ to obtain pressure of 1 bar in the *x*–*y* plane and the normal *z*‐direction with time constant of 5 ps. Then to equilibration, MD simulations were performed for 700 ns for each system; thus, a total of 2.8 μs has been performed in this work. In this step, the NPT ensemble was used, in the absence of restraints. In the simulations, LINCS algorithm was used to constrain bonds involving hydrogen.^85^ Electrostatic interactions were calculated using the particle mesh Ewald method^86^, ^87^ with cutoff of 1.2 Å, and van der Waals interactions used a cutoff of 12 Å. A time step of 2 fs was used for integration, while the coordinates and velocities were saved every 20 ps for analysis. Periodic boundary conditions were set in all directions.

### 4.2 Calculations of bilayer thickness and APL of DOPC bilayer

To assess the effect each polymorphic Aβ dimer on the DOPC bilayer properties, the values of APL and the bilayer thickness were computed for the peptide‐free DOPC bilayer and for the embedded Aβ dimer into the DOPC bilayer. These properties were calculated by using the grid‐based membrane analysis tool GridMAT‐MD.^88^ For the bilayer thickness, the phosphorus‐to‐phosphorus (P–P) distance was reported by using 20 grid‐point in *x* and *y* directions. The thickness values were calculated as averaged over 100 ns for the pure DOPC, and as averaged over 700 ns of each trajectory in which the conformations embedded within the DOPC, by using snapshots every 0.4 ns.

## AUTHOR CONTRIBUTIONS

**Olga Press‐Sandler:** Formal analysis (equal); investigation (equal); methodology (lead); resources (equal); validation (equal); visualization (lead); writing – original draft (equal); writing – review and editing (equal). **Yifat Miller:** Conceptualization (lead); formal analysis (equal); investigation (equal); project administration (lead); resources (equal); supervision (lead); validation (equal); writing – original draft (equal); writing – review and editing (equal).

## CONFLICT OF INTEREST

The authors declare that they have no conflicts of interest with the contents of this article.

## Supporting information


**Appendix S1**: Details of constructions of pure DOPC membrane, and Aβ dimers embedded in DOPC, analyses of the MD simulations, Tables S1 and S2, and Figures S1‐S18.
