# Supplementary material for: Molecular insights into the primary nucleation of polymorphic amyloid β dimers in DOPC lipid bilayer membrane
Source: Protein Sci. 2022 May;31(5):e4283. doi: 10.1002/pro.4283 (PMC8994488; doi:10.1002/pro.4283)
Supplement: Supplementary file 1 — Appendix S1: Details of constructions of pure DOPC membrane, and Aβ dimers embedded in DOPC, analyses of the MD simulations, Tables S1 and S2, and Figures S1‐S18. [file PRO-31-e4283-s001.docx]

Supporting Information for

**“Molecular insights into the primary nucleation of polymorphic amyloid β dimers in DOPC lipid bilayer membrane”**

Olga Press-Sandler, ^1,2^ Yifat Miller^1,2,*^

*^1^Department of Chemistry, Ben-Gurion University of the Negev, P.O. Box 653, Be'er Sheva 84105, Israel*

*^2^ Ilse Katz Institute for Nanoscale Science and Technology, Ben-Gurion University of the Negev, Beér-Sheva 84105, Israel*

Corresponding author:

Yifat Miller: [ymiller@bgu.ac.il](mailto:ymiller@bgu.ac.il)

**Methods and Materials**

**A construction of the pure 1,2-Dioleoyl-sn-glycero-3-phosphocholine (DOPC) bilayer membrane**

The phospholipids with phosphatidylcholine (PC) head groups are the most abundant lipids in neural membranes.^1^ Such vesicles are commonly used to investigate lipid raft domains, which have a fundamental biological function in the mammalian plasma membrane.^2^ Therefore, the DOPC bilayer was chosen in the current work to invesigate Aβ aggregates within this membrane model. In additon, the DOPC bilayer model was extensivly used both via experimental studies and computational studies for investigating Aβ aggregates in a channel-like model.^3-9^ The pure DOPC bilayer model was constructed by using CHARMM-GUI server.^10^ The constructed DOPC bilayer is composed of 184 lipids and was solvated with TIP3 water molecules and 100mM NaCl. The dimensions of the final solvated DOPC model were ~ 80 Å × 80 Å × 140 Å. Since the maximal widthess of Aβ dimer was ~ 50 Å, these dimensions of the bilayer offers diffusion distance of at least 10 Å along the xy membrane’s plane. The pure DOPC bilayer was subjected to 100 ns of molecular dynamics (MD) simulations and were applied as pre-equlibrated bilayer model for the insertion of each one of the polymorphic Aβ_1-42_ dimers.

**Constructions of Aβ_1-42_ dimers models within DOPC bilayer models**

The polymorphic Aβ_1-42_ dimers, models A1-A4, that were previously studied in solution^11^ were taken as initial models to the MD simulations within the DOPC bilayer membrane. The two “α-helix/random coil” dimers, model A1 and model A2, were constructed based on the solution NMR structure of Aβ_1-40_ monomer (PDB id code: 2LPM).^12^ An extention of two resideus at the C termini (I41 and A42) were preformed to constructe the full-length Aβ “α-helix/random coil” dimers. The two “α-helix/random coil” dimers, model A1 and model A2, were distinguished by the orientations of the two monomers within the dimer. The monomers in model A1 were arranged in parallel orientation, while in model A2 the monomers were arranged in antiparallel orientation. Yet, the interface between the two monomers within these two models considered the interactions between the central hydrophobic core (CHC) domain of each monomer (Figure S1).

An experimental study combined with MD simulations study showed that the N-truncated Aβ_9-42_ and Aβ_17-42_ peptides form fibrils with one U shape motif (i.e, β-strand-turn-β-strand) and produce toxic ion channels within DOPC bilayer.^6^ Obviously, one cannot expect that Aβ dimer will produce ion channels within DOPC bilayer. Yet, it is essential to investigate also early-stage dimers with a U shape “fibril-like” motif, that potentially may serve as a unit or a building block to the production of fibrils that eventually yield to ion channels within membranes. The two “fibril-like” dimers, models A3 and A4, were constructed based on the ssNMR structure of Aβ_17-42_ fibril (PDB id code: 2BEG).^13^ A monomer of Aβ_17-42_ was taken from the fibrillary structure and was elongated at the N-terminal residues D1-K16 to produce a full-length Aβ_1-42_ “fibril-like” monomer. The monomer was duplicated to dimers to construct the “fibril-like” parallel dimer- model A3 and the antiparallel “fibril-like” dimer - model A4 within DOPC bilayer (Figure S1).

Each one of the four models A1-A4 were firstly inserted into the pre-equlibrated DOPC bilayer by using the gmx membed program.^14^ Overlapping of lipids and water molecules were deleted in each model. In models A1, A3 and A4, the resideus K16 in each monomer were initally located at the water-lipid headgroup interface, and the residues L17-A42 were embedded within the DOPC hydrophobic core. In model A2, the residues E11 in each monomer were initally located at the lipid headgroup-water interface, and the residues V12-A42 were inserted within the DOPC hydrophobic core. Each DOPC-Aβ_1-42_ dimer was fully solvated by explicit TIP3 water molecules. The water molecules that were inserted inside the hydrohpbic core were removed from the DOPC bilayer by using in-house script (MATLAB). Finally, each DOPC-Aβ_1-42_ dimer was neutralized and ~100mM NaCl was added to create a physiological salt consentration.

We initially produced interactions between residues within two monomers that play role in the nucleation of Aβ aggregation, e.g., diphenylalanine F19-F20. The all-atom explicit MD simulations of these four constructed structures are costly, due to the relatively large biological systems. Therefore, the MD simulations approach limited us to investigate a limited number of polymorphic structures of Aβ dimers. Yet, we overcome this limitation by running relatively long timescale simulations (total of 2.8 μs for all four constructed structures). In fact, we observed in some cases a conformational change between several coformations. Along the MD simulation of each one of the four DOPC-Aβ_1-42_ dimers (models A1-A4), eight conformations were recognized. Conformations B1-B3 were identified in simulations of model A1. Conformation A2 was observed in simulations of model A2. Simulations of model A3 led to three conformations C1-C3, and model A4 led to one conformation A4. Table S1 summarizes the timescales in which each conformation was observed along the simulation of each DOPC-Aβ_1-42_ dimer model. The description of detection of these eight conformations are detailed as follows.

**Detection of conformations derived from the “random coil” Aβ dimers models**

In the parallel “α-helix/random coil” dimer (model A1) three conformations were recognized along the MD simulations: B1-B3 (Figure 1), according to their structural properties (Figure S2 and Figure S3). Three helices were conserved in these three conformations along the MD simulations (Figure S3): one helix in one monomer (residues K16-G25), and two helices in the second monomer (residues Q15-A21 and A30-G37). Interestingly, also previous studies of Aβ monomer demonstrated that the monomer has a helical structure and disordered regions in membranes and membrane-mimicking environments.^15-17^ Previously, MD simulations of Aβ_1-40_ dimer at different types of membranes (POPC, POPS, POPC/POPE and lipid Rafts) have also shown helical structures along residues Q15-D23 and K28-G38.^18^

While conformation B1 lacks a β-hairpin structural motif, in conformations B2 and B3 a short β-hairpin motif presents in one monomer within the dimer. In conformation B2, the β-hairpin is located at the C-terminal (β-strand: residues I32-G33, turn: residues L34-v36, and β-strand: G37-G38), while in conformation B3 the β-hairpin is placed at the N-terminal domain (residues β-strand: F4-H6, turn: residues D7-G9, and β-strand: Y10-V12). Previously, MD simulations of Aβ_1-40_ dimer at different types of membranes (POPC, POPS, POPC/POPE, Rafts and GM1-Rafts) have shown β-hairpin structure along the monomers at a similar N-terminal domain that observed in the current work (residues D1−Q15).^18^ In addition, MD simulations of Aβ_1-40_ dimer at GM1-Raft membrane demonstrated two β-hairpins that are located at the C-terminal domain (residues D23−A30 and residues A30−V40).^18^

In the antiparallel “α-helix/random coil” dimer, model A2, only one conformation was identified by the RMSDs analyses along the MD simulations: conformation A2 (Figure S4). One monomer consists of two helices that are located along residues G9-N27 and A30-V40, and the second monomer contains three helices that are positioned along residues G9-V12, F20-G25 and A30-L34 (Figure S5). This conformation lacks β-strands in both monomers within the dimer (Figure S5). In summary, all conformations that were derived from the two “α-helix/random coil” models indicate that the membrane environment does not trigger formation of β-strands between the two monomers that may lead to cross-β “fibril-like” structure. Thus, the “α-helix/random coil” dimers remain with properties of helical structures.

**Detection of conformations derived from the “fibril-like” Aβ dimers models**

In the parallel “fibril-like” dimer, model A3, three conformations were recognized along the MD simulations: conformations C1-C3 (Figure 1), due to the RMSDs analyses (Figure S6). Conformation C1 has a structural similarity to the U shape “fibril-like” structure. Conformation C2 is defined by a short β-hairpin structure at the N-terminal of one monomer and with a twisted “fibril-like” dimer. Finally, conformation C3 is recognized with two relatively long β-hairpin structures in one monomer: one at the N-terminal domain and the second at residues K16-V40. The second monomer is recognized with a longer β-hairpin structure at residues K16-V40 that wraps the second β-hairpin of the other monomer at the membrane DOPC core. Thus, in conformation C3, the two monomers form a “β-barrel-like” structure.

Interestingly, while the three-dimensional structure of each conformation is different, the secondary structural analysis indicates on a common β-strand properties along the sequence of both monomers within each one of the three conformations: One β-strand is located along the CHC domain (residues K16-A21), and the second β-strand encompass the SHC and C-terminal domains (residues I31-V40) (Figure S7). Finally, a structural change from a “α-helix/random coil” to “β-hairpin” was evolved only in one monomer at the N-terminal domain (residues A2-R5 and G9-V12) and was found in all three conformations (Figure S7).

Previously, MD simulations were performed for the “fibril-like” parallel Aβ_17-42_ dimer, that lacks the N-termini domains, in DOPC membrane.^19^ Interestingly, the initial “fibril-like” Aβ_17-42_ dimer that demonstrated well-organized cross-β structure was disrupted during the MD simulations in the DOPC bilayer membrane and β-hairpin structures were not produced. It has been shown that “fibril-like” Aβ_17-42_ pentamer conserves the cross-β sheet structure in the DOPC membrane but lacks β-hairpin structures. Thus, it was proposed that “fibril-like” Aβ_17-42_ trimer is the minimal oligomer size that may exhibit stable “fibril-like” Aβ_17-42_ structure. Moreover, it was suggested, though was not proven, that Aβ_17-42_ dimers may insert in “aggregation intermediate β-hairpin state” to the bilayer, and later may be converted (or not converted) to “fibril-like” U-shape structure.

Herein, the full length Aβ_1-42_ “fibril-like” dimer was investigated by MD simulations, and indeed, stable intermediate β-hairpin states were recognized. Moreover, the initial hydrophobic interactions that play role in Aβ_1-42_ fibrillation, were conserved along the MD simulations. Finally, our simulations reveal a formation of β-hairpin also at the N-terminal domain in one of the monomers, thus indicating the importance of the N-terminal domain to the stability of Aβ_1-42_ “fibril-like” dimer within the DOPC bilayer.

In the antiparallel “fibril-like” dimer - model A4, only one conformation was recognized along the MD simulation: conformation A4 (Figure 1), due to RMSDs analyses (Figure S8). Conformation A4 consists of two short parallel β-sheets: One β-sheet is located along residues K16-A21 of one monomer and along residues I31-V36 of the second monomer, while the second β-sheet is placed along residues K16-F20 of one monomer and along residues I31-M35 of the second monomer (Figure S9).

**Structural analysis details**

The structural stability of Aβ_1-42_ dimer models within the DOPC bilayer were analyzed by using root mean square deviation (RMSD) and the database of secondary structure of protein (DSSP) method. The convergences of the simulated models/conformations were produced by calculating the RMSD values of the backbone atoms for each Aβ_1-42_ dimer, using the ‘gmx rms’ GROMACS analysis tool. In the “α-helix/random coil” dimers, the RMSD values were calculated separately for two domains: N termini domains (residues D1-K16) and C-termini domains (residues L17-A42). In the “fibril-like” dimers, the RMSD values were calculated only for the ordered domains.

The secondary structure properties for each residues along the sequence of each monomer within the Aβ_1-42_ were estimated by using the DSSP method,^20^ by applying the ‘do dssp’ analysis in GROMACS. The representation of the secondary structure properties were classified into three groups: helix (helix/3^10^ helix/ π-helix), β strand/bridge, and turn/bend.

**Determining hydrophobic, electrostatic, and aromatic interactions between the peptides**

The interpeptide interactions were measured by the distances between atoms of residues along the MD simulations. All the distances were calculated by using ‘gmx distance’ in the GROMACS program. The hydrophobic interactions between two residues were estimated by measuring the distance between Cα atoms of two residues. The cutoff distance for hydrophobic interactions was set to 10 Å.^21^ The electrostatic interactions between two residues were estimated by the distance measurements between O atom of the acidic residue and the N atom of the base residue. The cutoff distance for electrostatic interactions was set to 4 Å.^22^ The π-π aromatic interactions were estimated by measuring the distance between C atoms of two aromatic residues. The cutoff distance for aromatic π-π interactions was set to 7 Å.^23^ The types of the C atoms of the aromatic residues that were applied to calculate the distances are detailed in Table S2. The reported percentage values are the averaged values of each two π-π interactions in Table S2.

**Interaction energy calculations between the DOPC lipids and Aβ_1-42_** **peptides**

To calculate the interaction energy values DOPC lipids and Aβ_1-42_ peptides between in each frame from the MD simulations, the ‘gmx energy’ program in the GROMACS package has been applied. The interaction energy values between each Aβ_1-42_ peptides and the DOPC lipids were computed for two types: electrostatic interactions (i.e., coulombic interactions), and VdW interactions (i.e., Lennard-Jones interactions).

**Calculations of the contacts between DOPC lipid headgroups and Aβ peptides**

The ‘gmx mindist’ GROMACS tool was applied to estimate the contacts between DOPC lipid headgroups and Aβ peptides. This tool is used to compute the minimum distance between each Aβ residue and the DOPC lipid headgroups. A contact was recorded when the distance between non-hydrogen atoms of each Aβ residue and the phosphors atoms of the lipid headgroups was within 4.5 Å. The number of counts were normalized between 0 to 1. The value 0 represents lack of contacts between DOPC lipid headgroups and Aβ peptide, and the value 1 indicates continuing contacts between DOPC lipid headgroups and Aβ peptide, along all time of the MD simulations. These calculations were previously applied to investigate the interactions between DOPC lipid headgroups and Aβ peptide for replica exchange molecular dynamics simulations of truncated Aβ_11-40_ trimer^24^ and mutation A21G in Aβ_11-40_ trimer.^25^

**Estimation of number of hydrogen bonds**

The averaged number of hydrogen bonds along 700 ns of the MD simulations were calculated between the Aβ_1-42_ monomers within the dimers and between Aβ_1-42_ dimers and the DOPC lipids. In the GROMACS ‘gmx hbond’ anlysis tool,^26^ a hydrogen bond is recorded when a donor (a hydrogen atom, that covalently binds to an electronegative atom) interacts with an acceptor (electronegative atom) by applying the following geometrical criteria:

1. r ≤ rHB= 3.5Å
2. α ≤ αhb =30$^{\circ}$

Where r is the distance between the donor and the acceptor atoms, and α is the angle between the acceptor and the donor atoms.

**
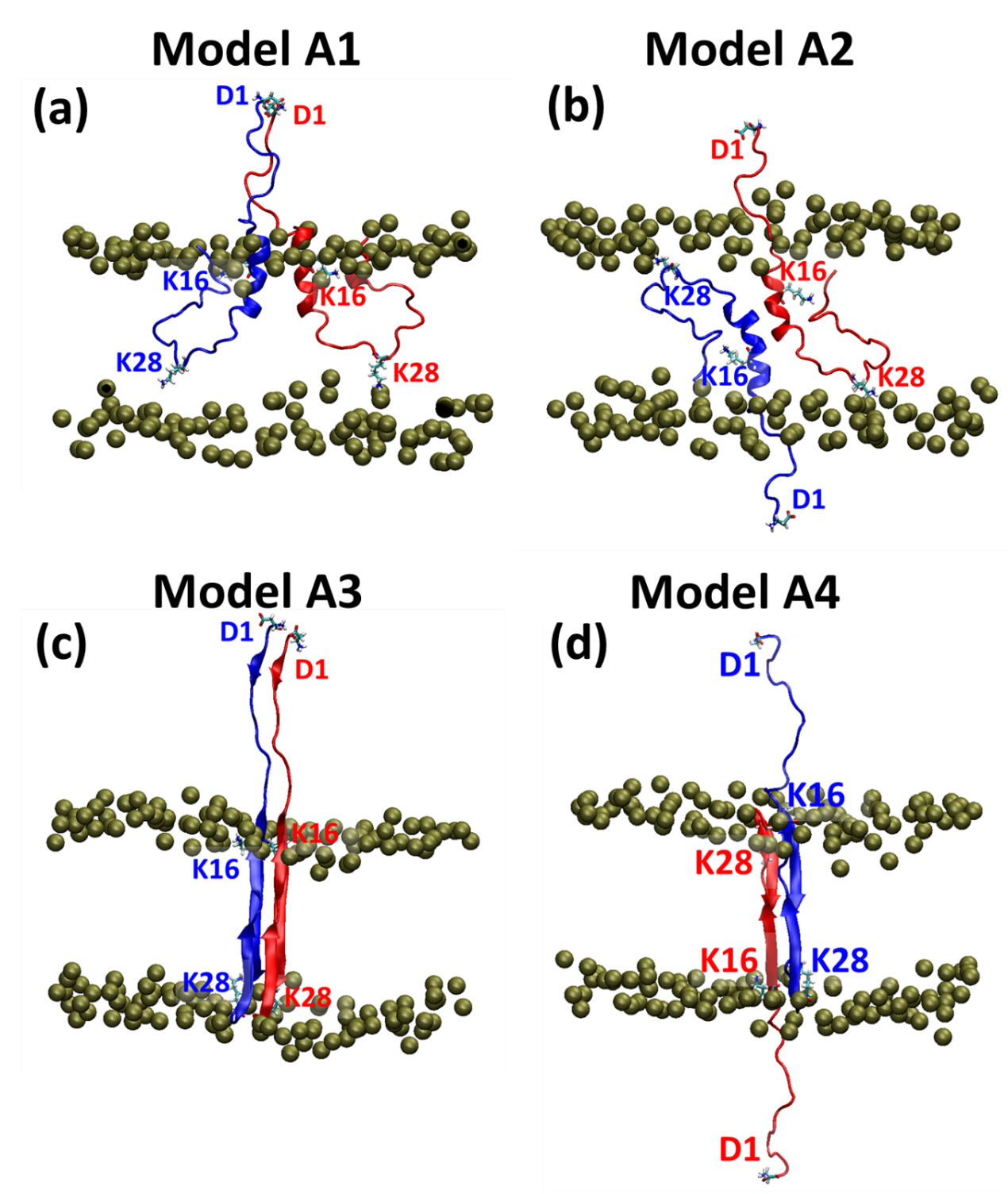
**

**Figure S1:** Initial constructed models of Aβ_1-42_ dimers within DOPC bilayer: (a) parallel “α-helix/random coil” dimer- model A1; (b) antiparallel “α-helix/random coil” dimer- model A2; (c) parallel “fibril-like” dimer- model A3; (d) antiparallel “fibril-like” dimer- model A4. The Aβ monomers are colored in red and blue. The phosphorus atoms of the DOPC lipid headgroups are shown in Van der Waals spheres. The acyl chains are not shown for clarity.

**
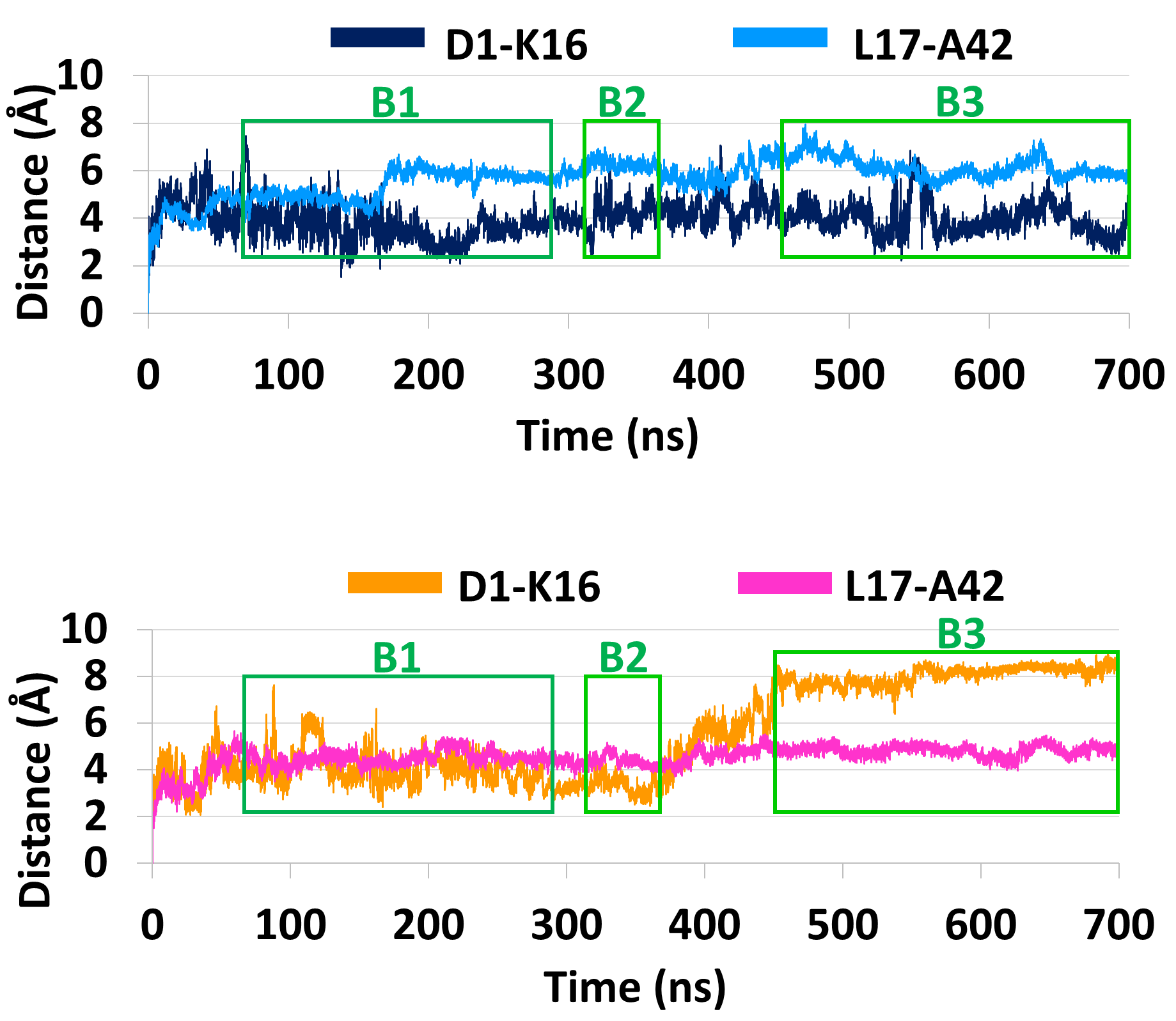
**

**Figure S2:** The RMSDs of one Aβ monomer (top) and the second Aβ monomer (bottom) along the MD simulations of model A1, for two sequences along Aβ: D1-K16 and L17-A42. Conformations B1-B3 were recognized along the MD simulations.

**
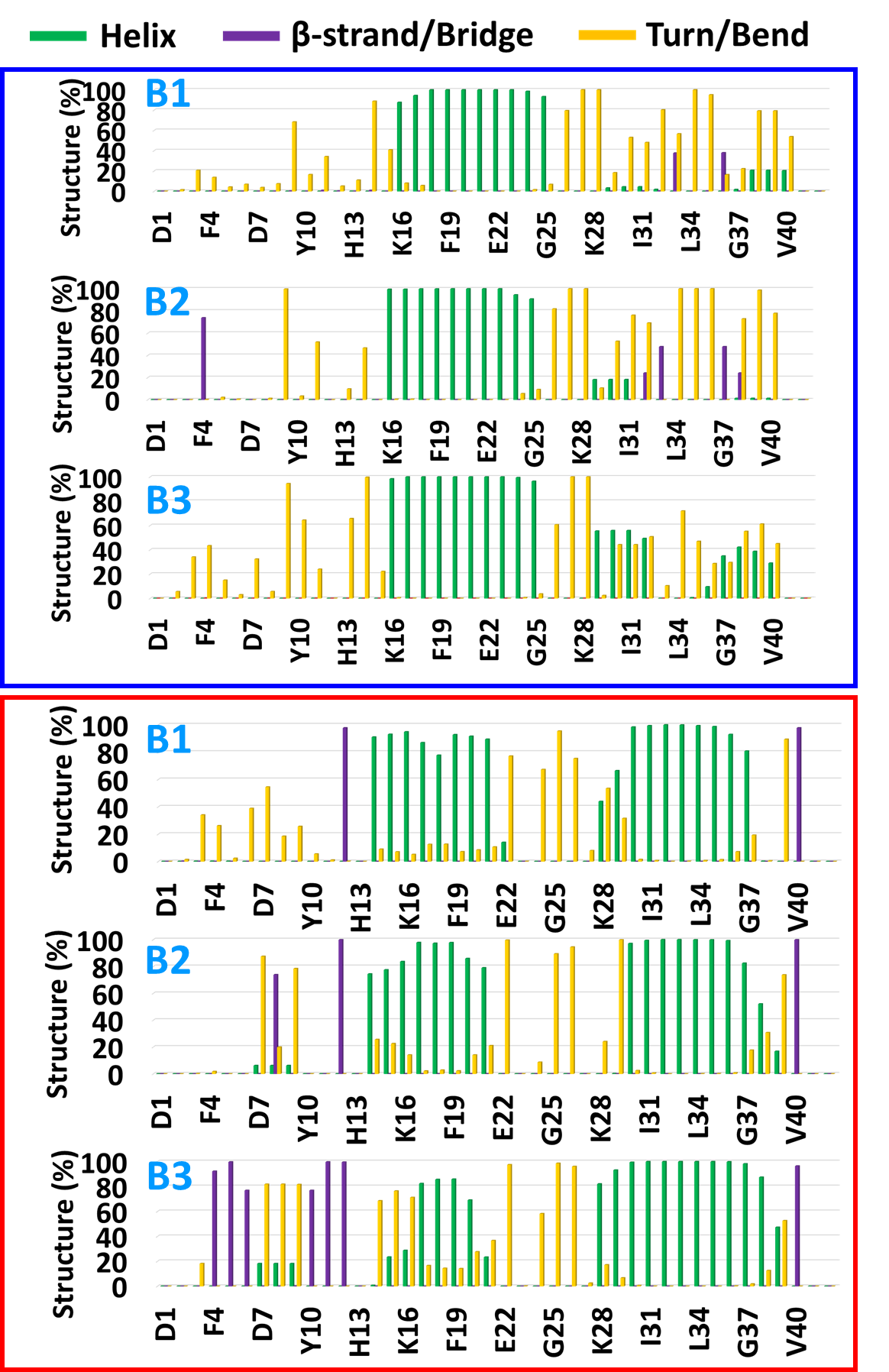
**

**Figure S3:** Secondary structure along the sequence of one Aβ monomer (top) and the second Aβ monomer (bottom) for conformations B1-B3.

**
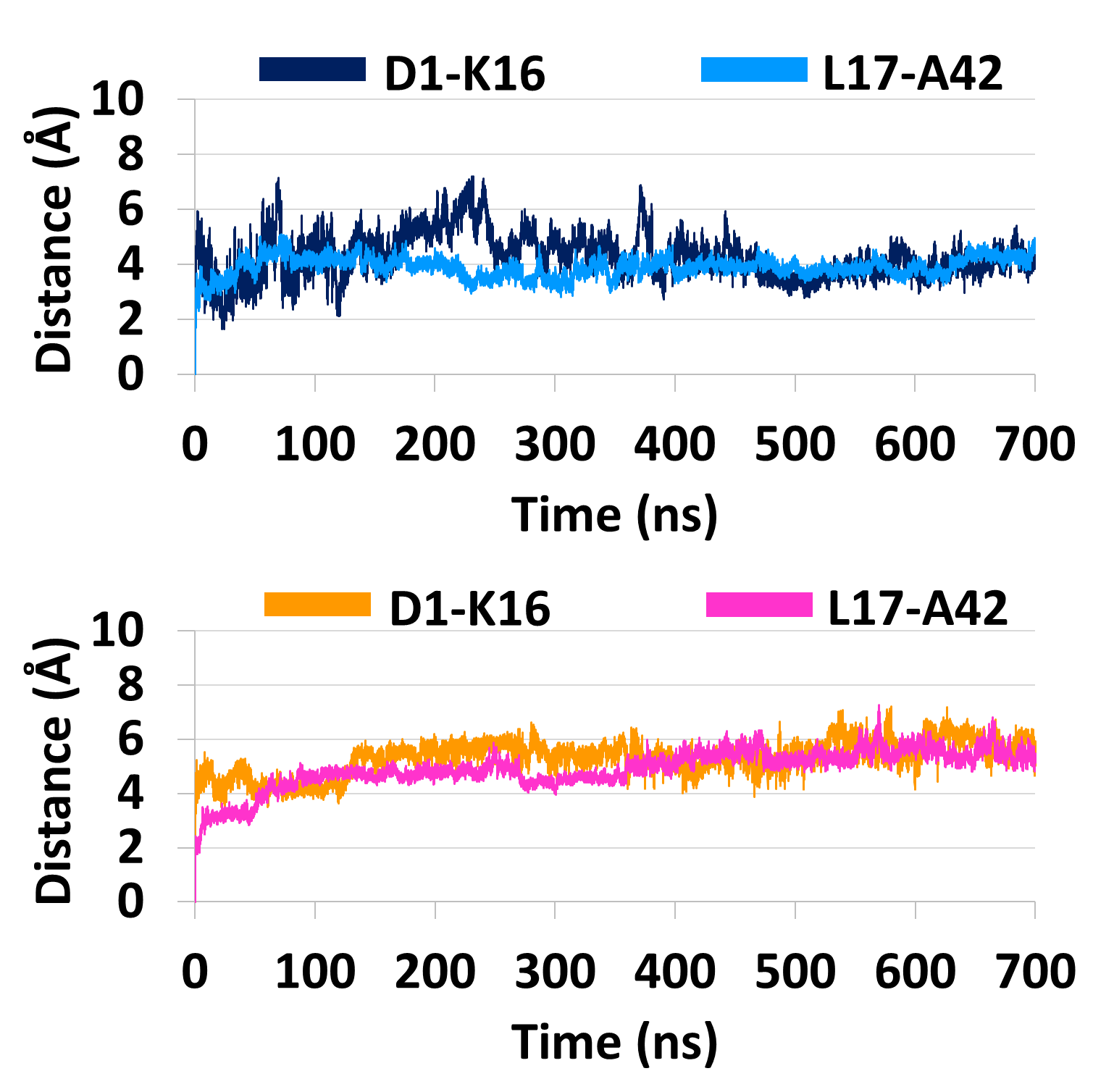
**

**Figure S4:** The RMSDs of one Aβ monomer (top) and the second Aβ monomer (bottom) along the MD simulations of conformation A2, for two sequences along Aβ: D1-K16 and L17-A42.

**
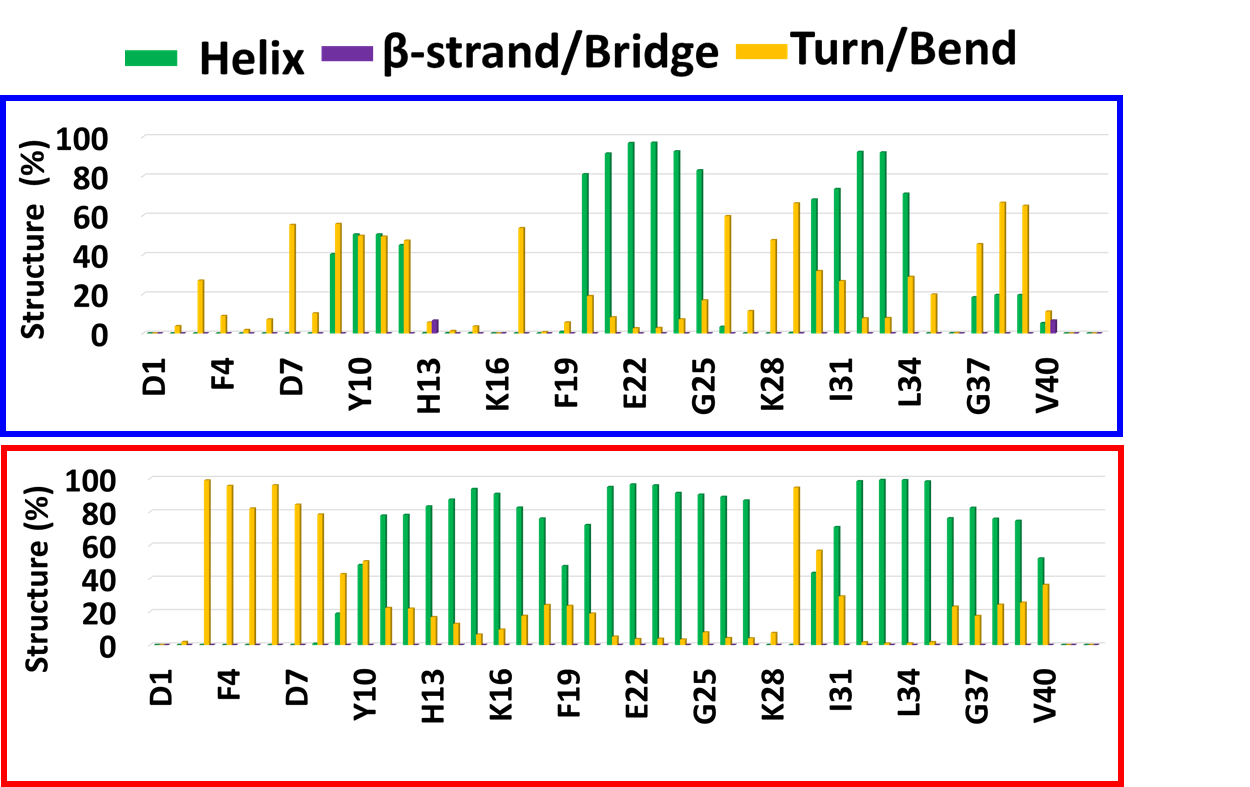
**

**Figure S5:** Secondary structure along the sequence of one Aβ monomer (top) and the second Aβ monomer (bottom) for conformation A2.


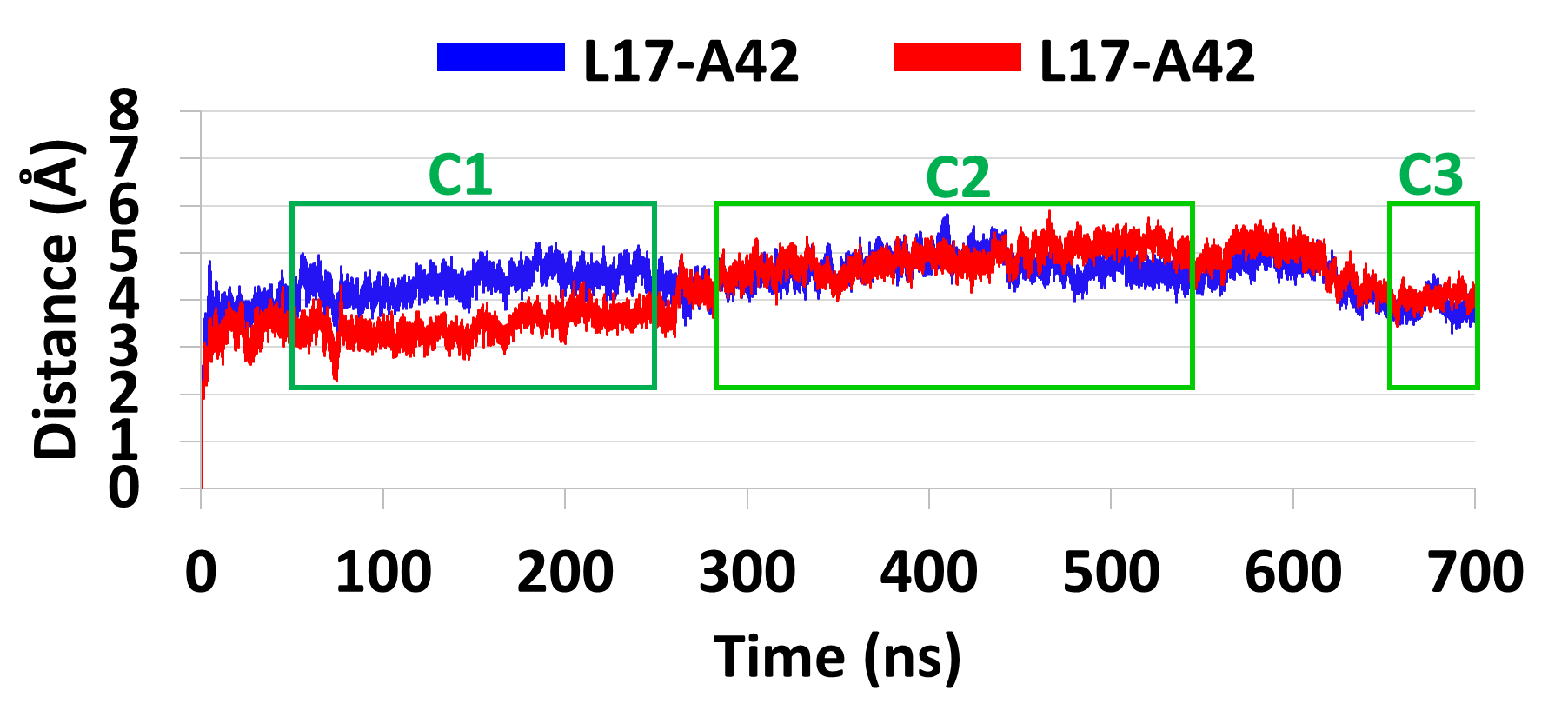


**Figure S6:** The RMSDs of each Aβ monomer (colors: blue and red) along the MD simulations of model A3 for residues L17-A42. Conformations C1-C3 were recognized along the MD simulations.

**
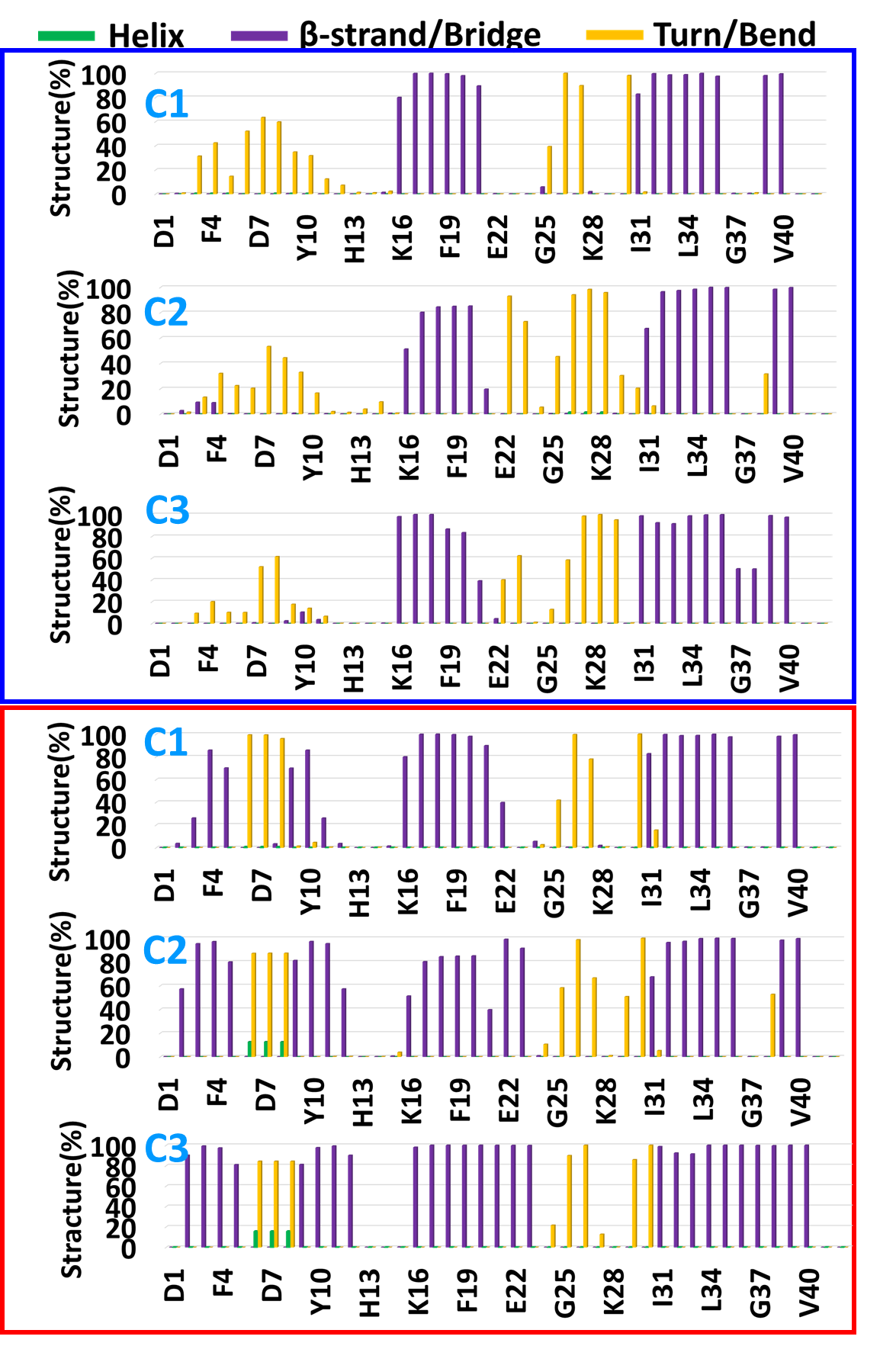
**

**Figure S7:** Secondary structure along the sequence of one Aβ monomer (top) and the second Aβ monomer (bottom) for conformations C1-C3.


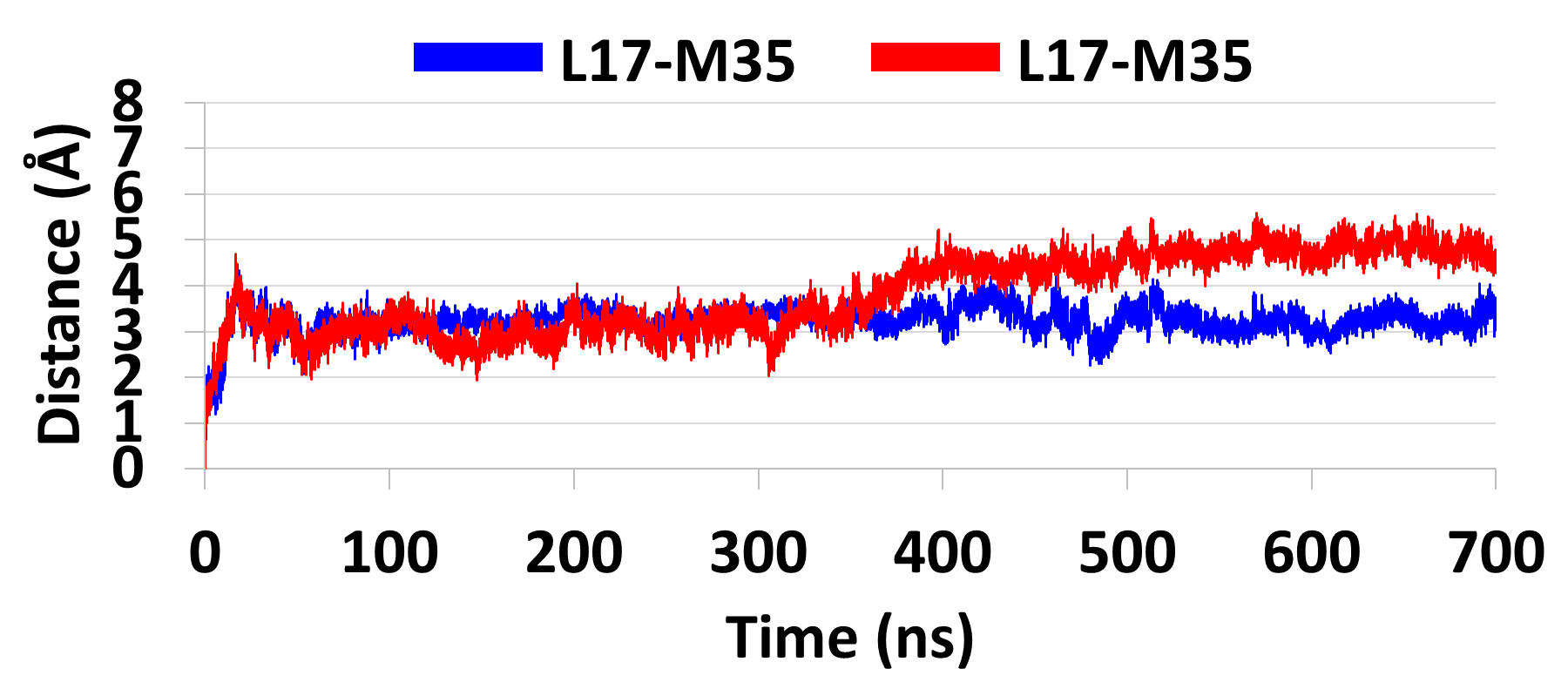


**Figure S8:** The RMSDs of each Aβ monomer (colors: blue and red) along the MD simulations of conformation A4 for residues L17-A35. Residues D1-K16 and V36-A42 were excluded.


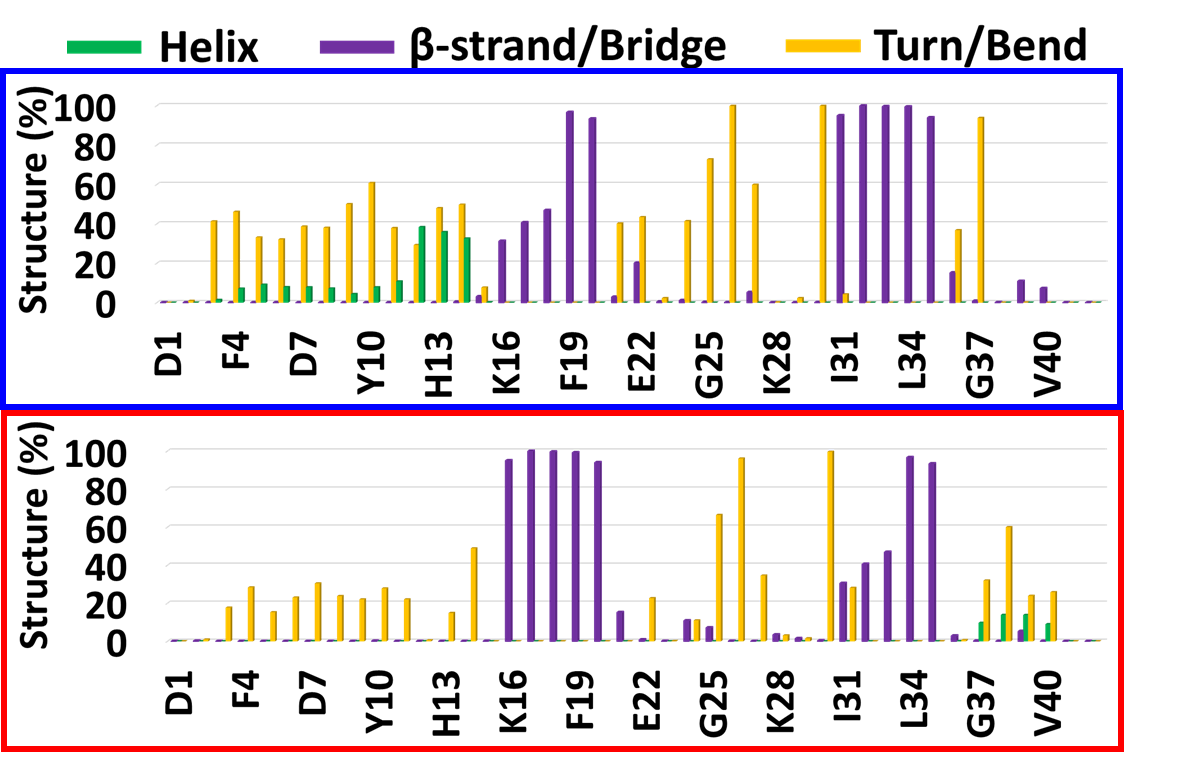


**Figure S9:** Secondary structure along the sequence of one Aβ monomer (top) and the second Aβ monomer (bottom) for conformation A4.


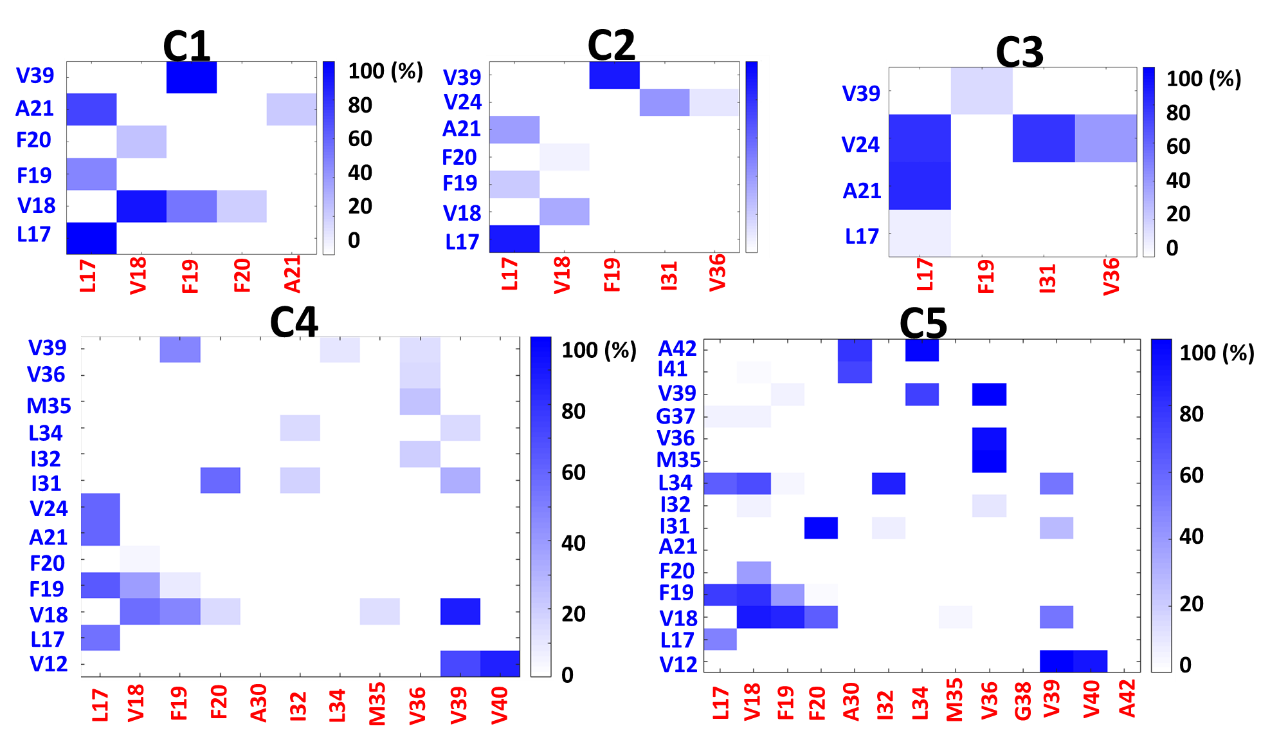


**Figure S10:** Percentage of hydrophobic interactions between Aβ monomers within the dimer for conformations C1-C5 that derived from simulated model A1 in solution. The residues in the contact maps are colored in red and blue and represent the residues of the two Aβ monomers**.** Data taken from Ref. 11.


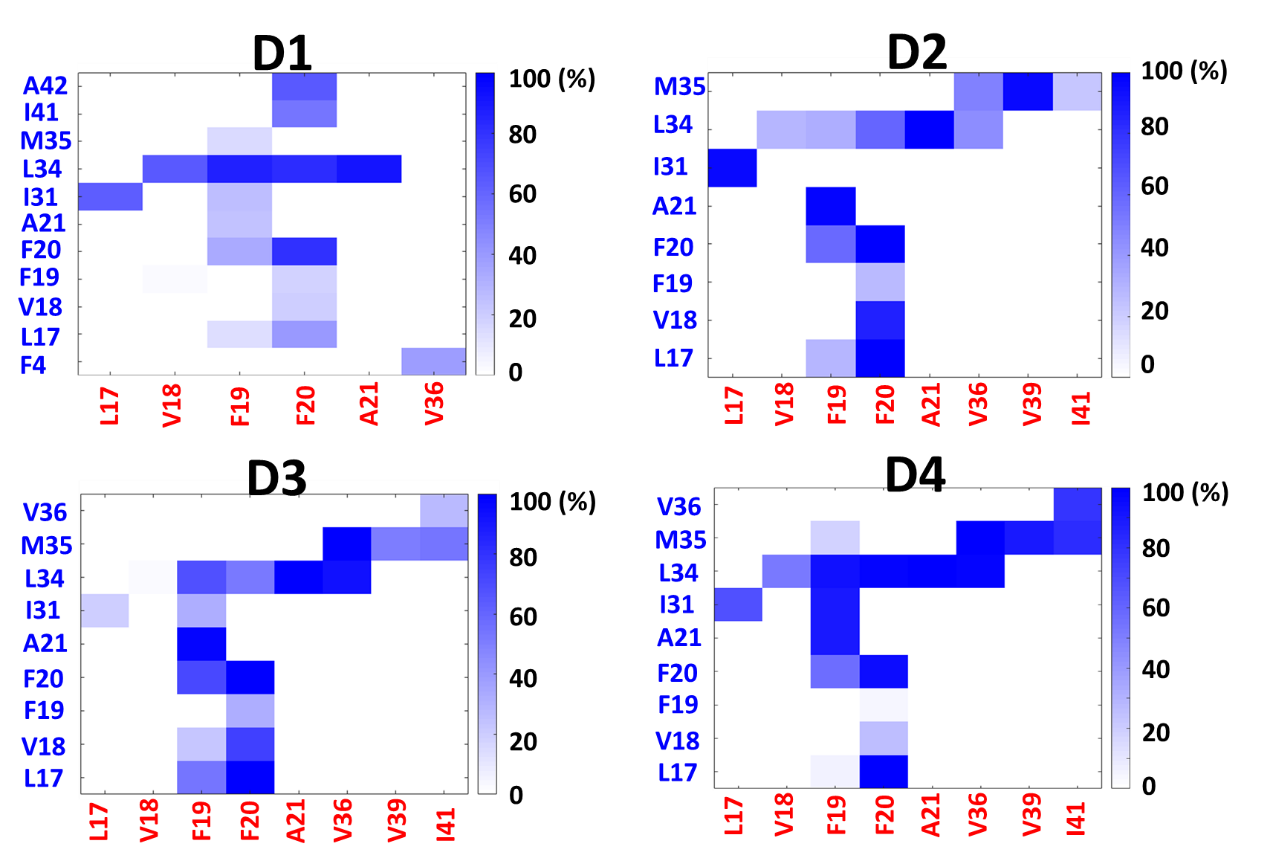


**Figure S11:** Percentage of hydrophobic interactions between Aβ monomers within the dimer for conformations D1-D4 that derived from simulated model A2 in solution. The residues in the contact maps are colored in red and blue and represent the residues of the two Aβ monomers**.** Data taken from Ref. 11.


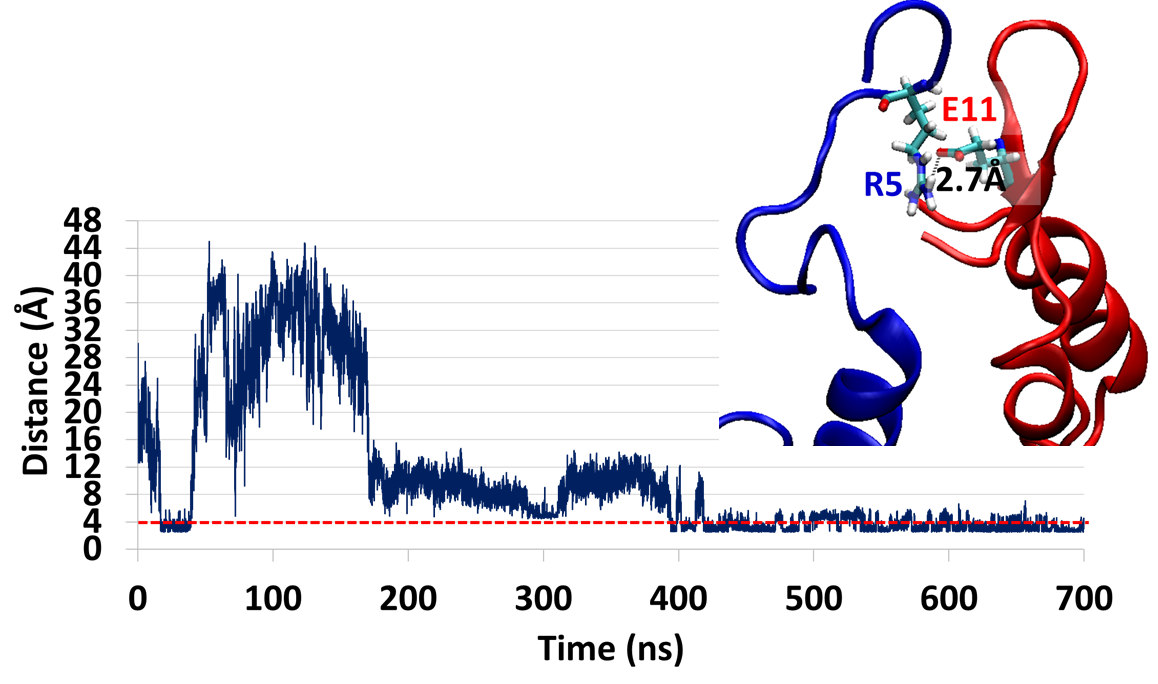


**Figure S12:** The distance between O atom in the anionic carboxylate of E11 in one Aβ monomer (color: red) and the N atom in the [guanidinium](https://en.wikipedia.org/wiki/Guanidine) of R5 in the second Aβ (color: blue) along the MD simulations. The measured R5-E11 salt-bridge interactions in conformations B1-B3. The snapshot was taken from the MD simulations of conformation B3.


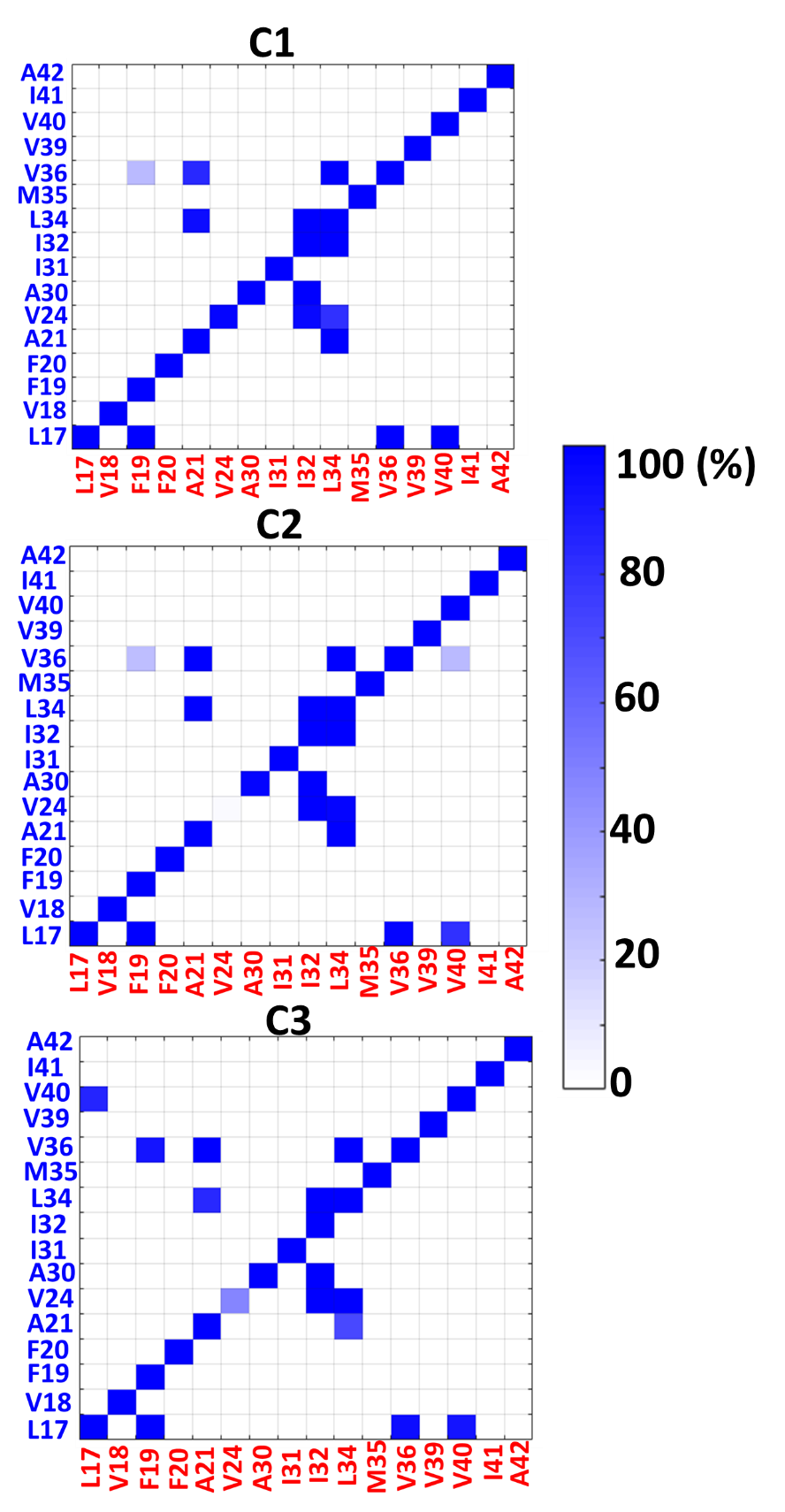


**Figure S13:** Percentage of hydrophobic interactions between Aβ monomers within the dimer for separated conformations C1-C3. The residues in the contact maps are colored in red and blue and represent the residues of the two Aβ monomers.


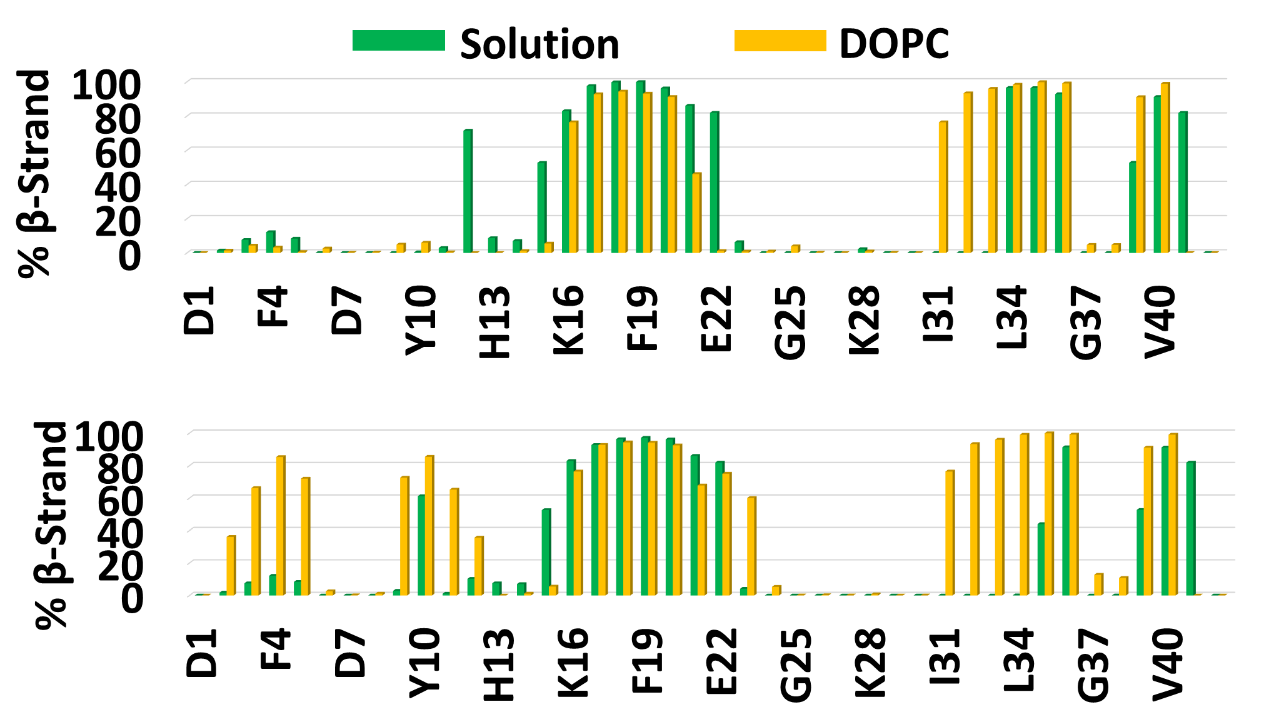


**Figure S14:** Secondary structure of β-strands propensity of one Aβ monomer (top) and second Aβ monomer (bottom) of the simulated model A3 in solution, and of the simulated model A3 in DOPC, i.e., the total conformations C1-C3.


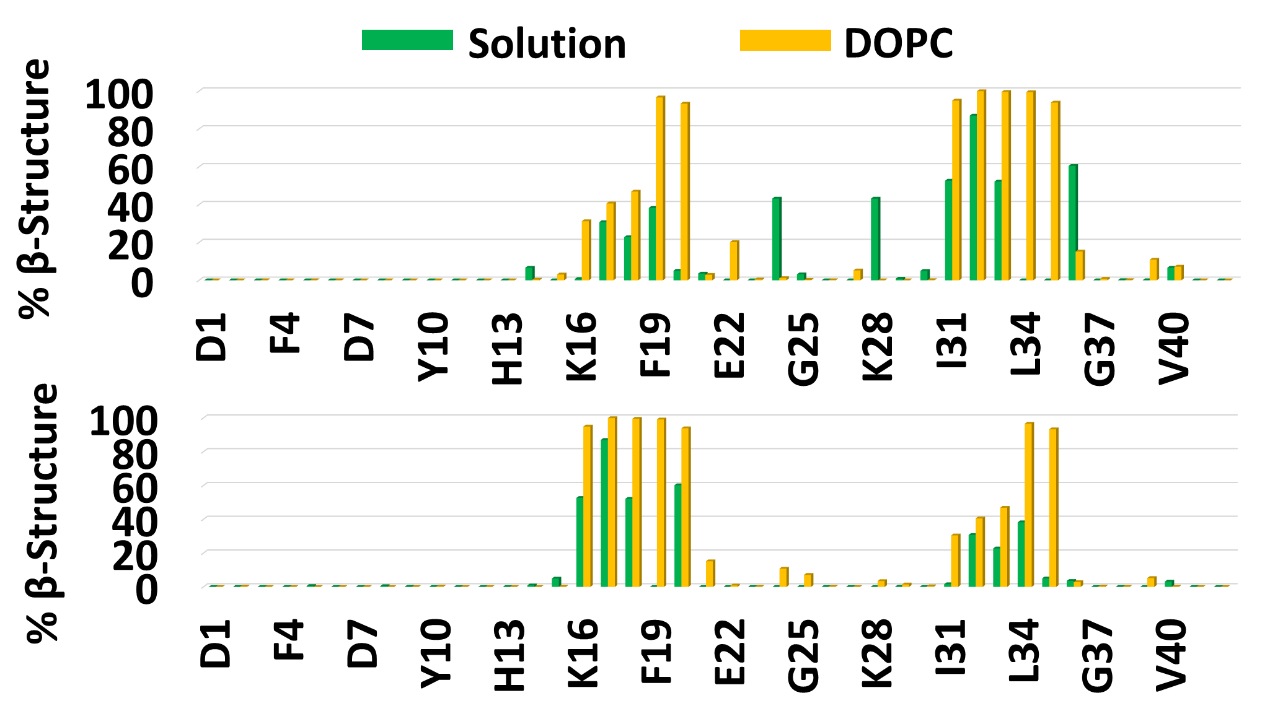


**Figure S15:** Secondary structure of β-strands propensity of one Aβ monomer (top) and second Aβ monomer (bottom) of the simulated model A4 in solution, and of the simulated model A4 in DOPC, i.e., conformation A4.


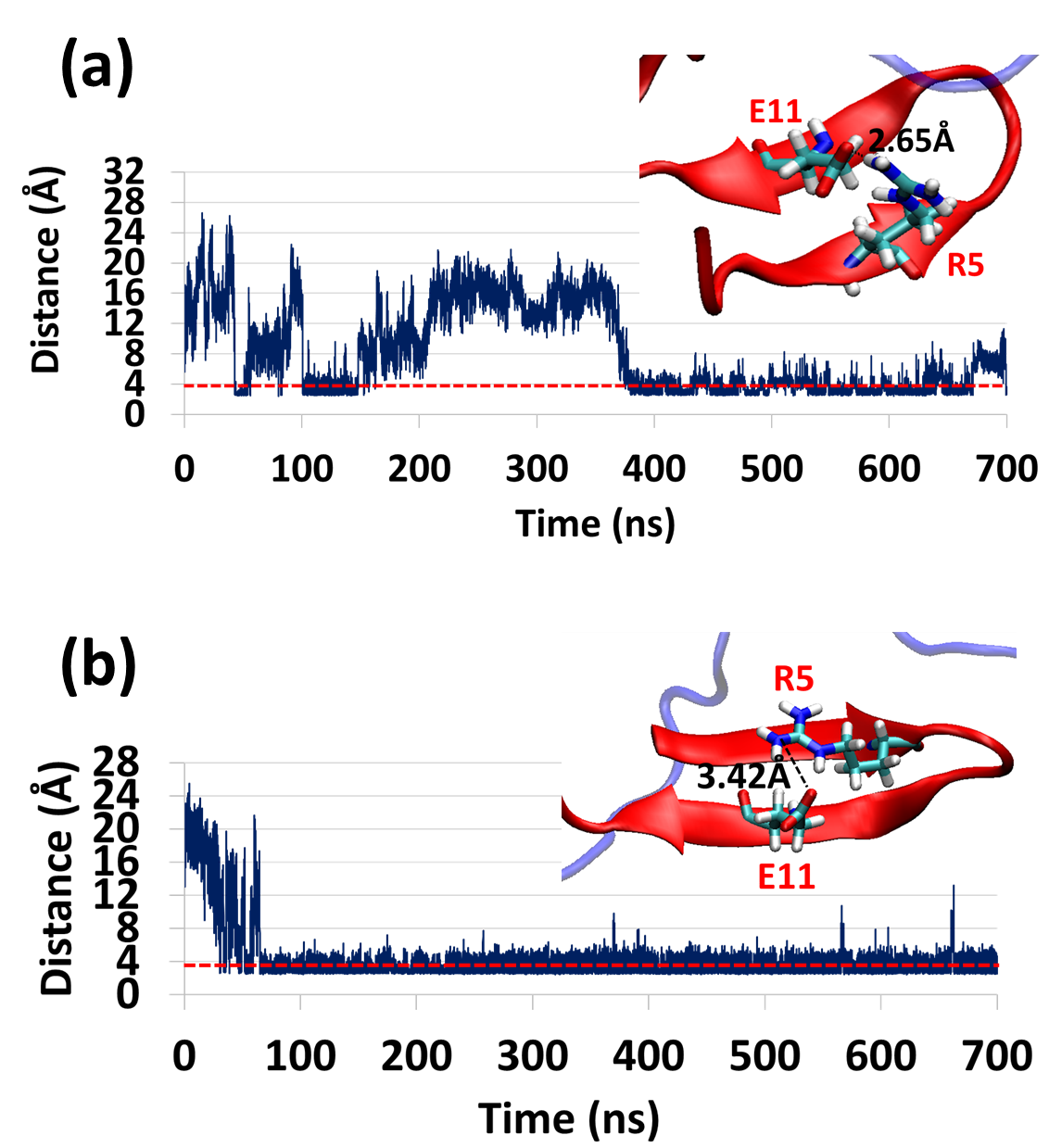


**Figure S16:** The distance between O atom in the anionic carboxylate of E11 and the N atom in the [guanidinium](https://en.wikipedia.org/wiki/Guanidine) of R5 along the MD simulations for: (a) conformations B1-B3, and (b) conformations C1-C3. The measured R5-E11 salt-bridge interactions were taken from snapshots from MD simulations. The red dashed line represents the cutoff distance of electrostatic interactions.


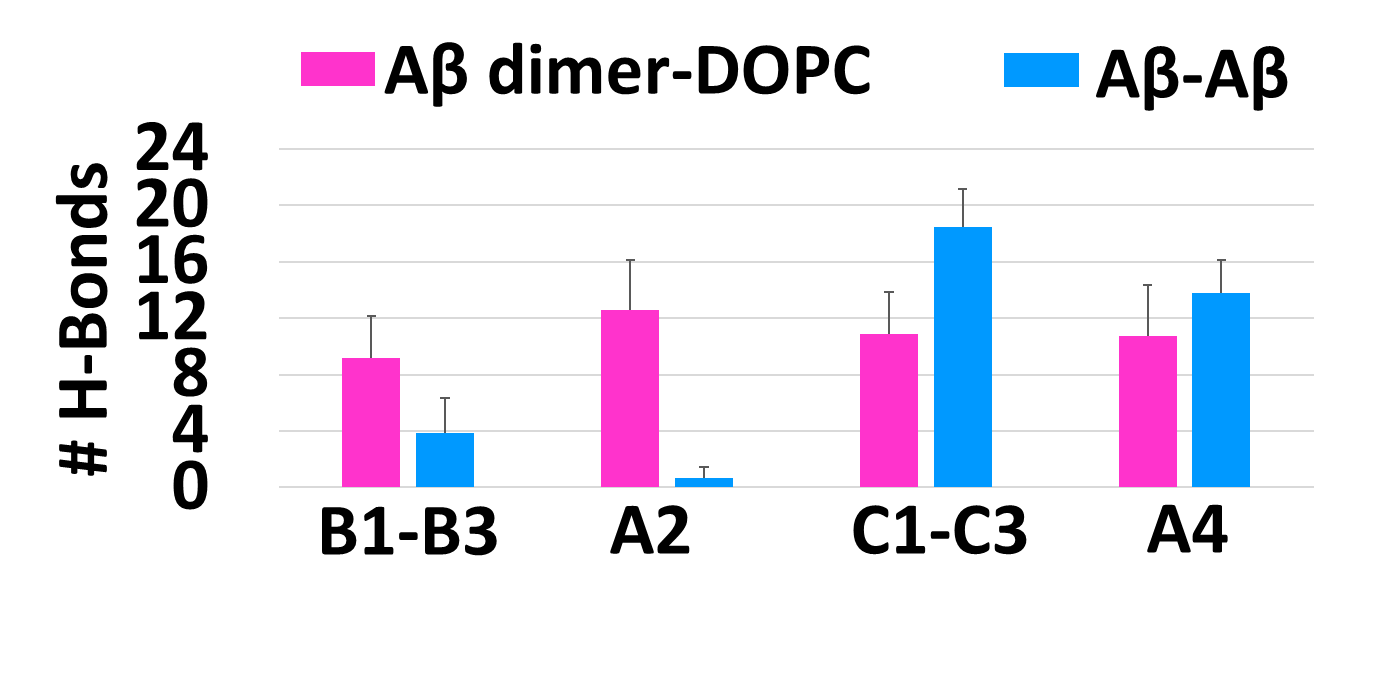


**Figure S17:** An averaged number of hydrogen bond interactions between Aβ dimers and the DOPC lipids, and between Aβ monomers within the dimers for all eight conformations.


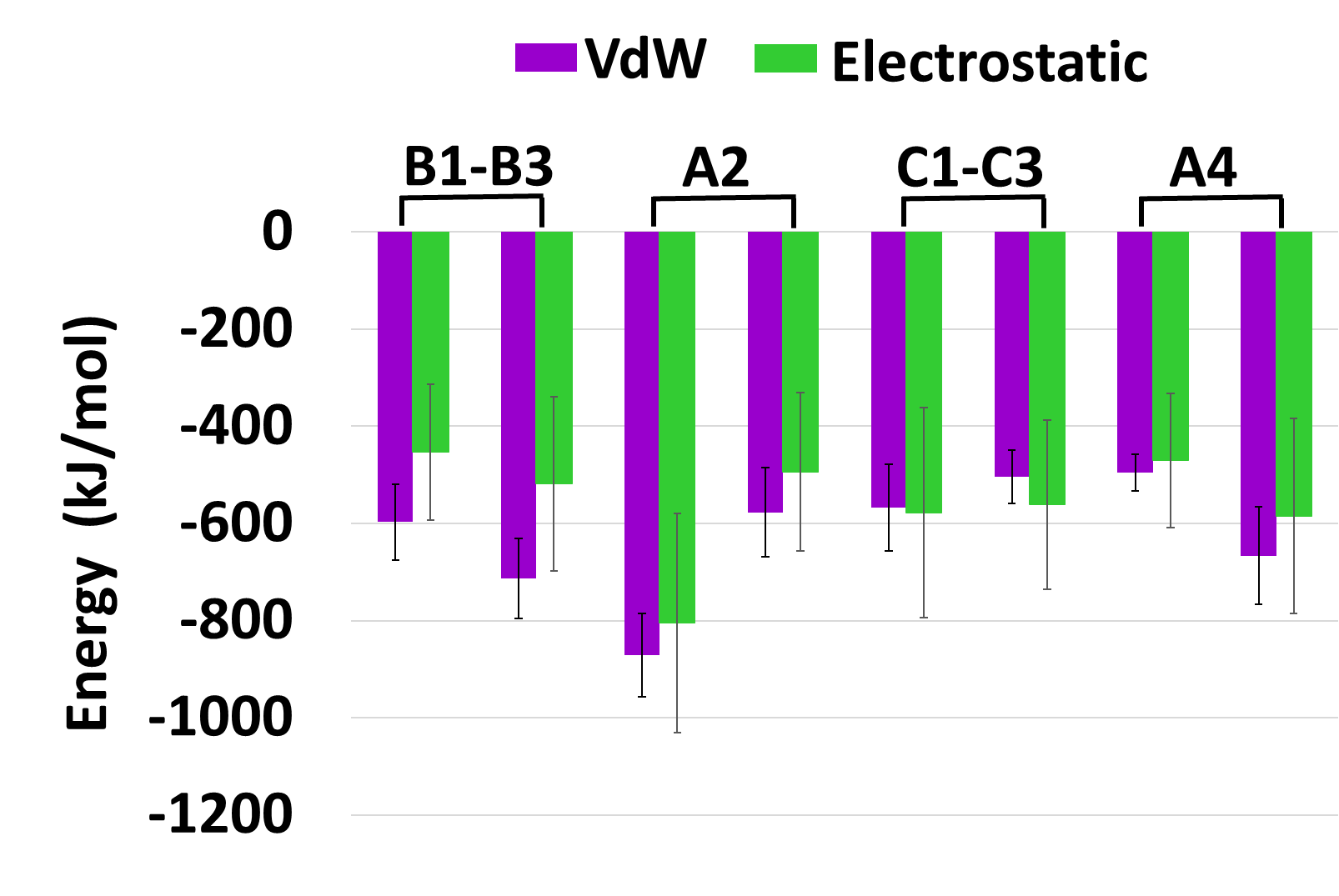


**Figure S18:** An averaged energy of VdW and electrostatic interactions between each Aβ monomer and the DOPC lipids, for all eight conformations. The energies were calculated for each Aβ monomer: histograms in left are calculated values for one monomer, and histograms in right are calculated values for second monomer.

**Table S1**: The timescales of the identification of each conformation along the MD simulations of the each model.

| Model | Conformation | Timescale along MD simulations (ns) |
| --- | --- | --- |
| A1 | B1 | 50-300 |
|  | B2 | 330-360 |
|  | B3 | 450-700 |
| A2 | A2 | 0-700 |
| A3 | C1 | 65-250 |
|  | C2 | 300-550 |
|  | C3 | 650-700 |
| A4 | A4 | 0-700 |

**Table S2**: The types of the C atoms of F19 and F20 in each Aβ monomer (monomer 1: color: blue; monomer 2: color: red) that were applied to measure the distances that estimate the aromatic π-π interactions.

| Model | Resideue in Aβ | Atom in Aβ | Residue in Aβ | Atom in Aβ |
| --- | --- | --- | --- | --- |
| A1 | F20 | Cγ | F20 | Cζ |
|  |  | Cγ |  | Cε2 |
| A2 | F19 | Cζ | F20 | Cε1 |
|  |  | Cε1 |  | Cζ |
|  | F20 | Cδ1 | F20 | Cδ1 |
|  |  | Cγ |  | Cε1 |
| A3 | F19 | Cδ2 | F19 | Cδ2 |
|  |  | Cδ1 |  | Cδ1 |
|  | F20 | Cδ1 | F20 | Cδ1 |
|  |  | Cε1 |  | Cε1 |

**References**

1. Norton, W. T.; Abe, T.; Poduslo, S. E.; Devries, G. H., Lipid-Composition of Isolated Brain-Cells and Axons. *Journal of Neuroscience Research* 1975, 1, 57-75.

2. Luchini, A.; Vitiello, G., Mimicking the Mammalian Plasma Membrane: An Overview of Lipid Membrane Models for Biophysical Studies. *Biomimetics* 2021, 6.

3. Quist, A.; Doudevski, I.; Lin, H.; Azimova, R.; Ng, D.; Frangione, B.; Kagan, B.; Ghiso, J.; Lal, R., Amyloid ion channels: a common structural link for protein-misfolding disease. *Proc Natl Acad Sci U S A* 2005, 102, 10427-32.

4. Connelly, L.; Jang, H.; Arce, F. T.; Capone, R.; Kotler, S. A.; Ramachandran, S.; Kagan, B. L.; Nussinov, R.; Lal, R., Atomic force microscopy and MD simulations reveal pore-like structures of all-D-enantiomer of Alzheimer's beta-amyloid peptide: relevance to the ion channel mechanism of AD pathology. *J Phys Chem B* 2012, 116, 1728-35.

5. Jang, H.; Arce, F. T.; Capone, R.; Ramachandran, S.; Lal, R.; Nussinov, R., Misfolded amyloid ion channels present mobile beta-sheet subunits in contrast to conventional ion channels. *Biophys J* 2009, 97, 3029-37.

6. Jang, H.; Arce, F. T.; Ramachandran, S.; Capone, R.; Azimova, R.; Kagan, B. L.; Nussinov, R.; Lal, R., Truncated beta-amyloid peptide channels provide an alternative mechanism for Alzheimer's Disease and Down syndrome. *Proc Natl Acad Sci U S A* 2010, 107, 6538-43.

7. Jang, H.; Arce, F. T.; Ramachandran, S.; Kagan, B. L.; Lal, R.; Nussinov, R., Familial Alzheimer's disease Osaka mutant (DeltaE22) beta-barrels suggest an explanation for the different Abeta1-40/42 preferred conformational states observed by experiment. *J Phys Chem B* 2013, 117, 11518-29.

8. Jang, H.; Arce, F. T.; Ramachandran, S.; Kagan, B. L.; Lal, R.; Nussinov, R., Disordered amyloidogenic peptides may insert into the membrane and assemble into common cyclic structural motifs. *Chem Soc Rev* 2014, 43, 6750-64.

9. Jang, H.; Zheng, J.; Nussinov, R., Models of beta-amyloid ion channels in the membrane suggest that channel formation in the bilayer is a dynamic process. *Biophys J* 2007, 93, 1938-49.

10. Jo, S.; Kim, T.; Iyer, V. G.; Im, W., CHARMM-GUI: a web-based graphical user interface for CHARMM. *J Comput Chem* 2008, 29, 1859-65.

11. Press-Sandler, O.; Miller, Y., Distinct Primary Nucleation of Polymorphic Abeta Dimers Yields to Distinguished Fibrillation Pathways. *ACS Chem Neurosci* 2019, 10, 4407-4413.

12. Vivekanandan, S.; Brender, J. R.; Lee, S. Y.; Ramamoorthy, A., A partially folded structure of amyloid-beta(1-40) in an aqueous environment. *Biochem Biophys Res Commun* 2011, 411, 312-6.

13. Luhrs, T.; Ritter, C.; Adrian, M.; Riek-Loher, D.; Bohrmann, B.; Dobeli, H.; Schubert, D.; Riek, R., 3D structure of Alzheimer's amyloid-beta(1-42) fibrils. *Proc Natl Acad Sci U S A* 2005, 102, 17342-7.

14. Wolf, M. G.; Hoefling, M.; Aponte-Santamaria, C.; Grubmuller, H.; Groenhof, G., g_membed: Efficient insertion of a membrane protein into an equilibrated lipid bilayer with minimal perturbation. *J Comput Chem* 2010, 31, 2169-74.

15. Coles, M.; Bicknell, W.; Watson, A. A.; Fairlie, D. P.; Craik, D. J., Solution structure of amyloid beta-peptide(1-40) in a water-micelle environment. Is the membrane-spanning domain where we think it is? *Biochemistry* 1998, 37, 11064-77.

16. Crescenzi, O.; Tomaselli, S.; Guerrini, R.; Salvadori, S.; D'Ursi, A. M.; Temussi, P. A.; Picone, D., Solution structure of the Alzheimer amyloid beta-peptide (1-42) in an apolar microenvironment. Similarity with a virus fusion domain. *Eur J Biochem* 2002, 269, 5642-8.

17. Sticht, H.; Bayer, P.; Willbold, D.; Dames, S.; Hilbich, C.; Beyreuther, K.; Frank, R. W.; Rosch, P., Structure of amyloid A4-(1-40)-peptide of Alzheimer's disease. *Eur J Biochem* 1995, 233, 293-8.

18. Lemkul, J. A.; Bevan, D. R., Aggregation of Alzheimer's amyloid beta-peptide in biological membranes: a molecular dynamics study. *Biochemistry* 2013, 52, 4971-80.

19. Jang, H.; Connelly, L.; Arce, F. T.; Ramachandran, S.; Kagan, B. L.; Lal, R.; Nussinov, R., Mechanisms for the Insertion of Toxic, Fibril-like beta-Amyloid Oligomers into the Membrane. *J Chem Theory Comput* 2013, 9, 822-833.

20. Kabsch, W.; Sander, C., Dictionary of protein secondary structure: pattern recognition of hydrogen-bonded and geometrical features. *Biopolymers* 1983, 22, 2577-637.

21. Israelachvili, J.; Pashley, R., The hydrophobic interaction is long range, decaying exponentially with distance. *Nature* 1982, 300, 341-2.

22. Kumar, S.; Nussinov, R., Close-range electrostatic interactions in proteins. *Chembiochem* 2002, 3, 604-17.

23. Burley, S. K.; Petsko, G. A., Aromatic-aromatic interaction: a mechanism of protein structure stabilization. *Science* 1985, 229, 23-8.

24. Ngo, S. T. H., H. M.; Tran, K. N.; Nguyen, M. T., Replica Exchange Molecular Dynamics Study of the Amyloid Beta (11−40) Trimer Penetrating a Membrane. *RSC Adv.* 2017, 7, 7346−7357.

25. Ngo, S. T. N., M.T.; Nguyen, N.T. ;Vu, V.V., The Effects of A21G Mutation on Transmembrane Amyloid Beta (11-40) Trimer: An In Silico Study. *J Phys Chem B* 2017, 121, 8467-8474.

26. Abraham, M. J. M., T.; Schulz, R.; Páll, S.; Smith, J.C.; Hess, B.; Lindahl, E., GROMACS: High performance molecular simulations through multi-level parallelism from laptops to supercomputers. *SoftwareX* 2015, 1-2, 19-25.
